# Silica Triggers Inflammation and Ectopic Lymphoid Neogenesis in the Lungs in Parallel with Accelerated Onset of Systemic Autoimmunity and Glomerulonephritis in the Lupus-Prone NZBWF1 Mouse

**DOI:** 10.1371/journal.pone.0125481

**Published:** 2015-05-15

**Authors:** Melissa A. Bates, Christina Brandenberger, Ingeborg Langohr, Kazuyoshi Kumagai, Jack R. Harkema, Andrij Holian, James J. Pestka

**Affiliations:** 1 Department of Food Science and Human Nutrition, Michigan State University, East Lansing, Michigan, United States of America; 2 Center for Integrative Toxicology, Michigan State University, East Lansing, Michigan, United States of America; 3 Department of Pathobiology and Diagnostic Investigation, Michigan State University, East Lansing, Michigan, United States of America; 4 Functional and Applied Anatomy, Hannover Medical School, Hannover, Germany; 5 Department of Pathobiological Studies, School of Veterinary Medicine, Louisiana State University, Baton Rogue, Louisiana, United States of America; 6 Center for Environmental Health Sciences, University of Montana, Missoula, Montana, United States of America; Instituto Nacional de Ciencias Medicas y Nutricion Salvador Zubiran, MEXICO

## Abstract

Genetic predisposition and environmental factors influence the development of human autoimmune disease. Occupational exposure to crystalline silica (*c*SiO_2_) has been etiologically linked to increased incidence of autoimmunity, including systemic lupus erythematosus (SLE), but the underlying mechanisms are poorly understood. The purpose of this study was to test the hypothesis that early repeated short-term *c*SiO_2_ exposure will modulate both latency and severity of autoimmunity in the lupus-prone female NZBWF1 mouse. Weekly intranasal exposure to *c*SiO_2_ (0.25 and 1.0 mg) for 4 wk beginning at 9 wk of age both reduced latency and increased intensity of glomerulonephritis. *c*SiO_2_ elicited robust inflammatory responses in the lungs as evidenced by extensive perivascular and peribronchial lymphoplasmacytic infiltration consisting of IgG-producing plasma cells, and CD45R+ and CD3+ lymphocytes that were highly suggestive of ectopic lymphoid tissue (ELT). In addition, there were elevated concentrations of immunoglobulins and the cytokines MCP-1, TNF-α and IL-6 in bronchoalveolar lavage fluid. *c*SiO_2_-associated kidney and lung effects paralleled dose-dependent elevations of autoantibodies and proinflammatory cytokines in plasma. Taken together, *c*SiO_2_-induced pulmonary inflammation and ectopic lymphoid neogenesis in the NZBWF1 mouse corresponded closely to systemic inflammatory and autoimmune responses as well as the early initiation of pathological outcomes in the kidney. These findings suggest that following airway exposure to crystalline silica, in mice genetically prone to SLE, the lung serves as a platform for triggering systemic autoimmunity and glomerulonephritis.

## Introduction

Development of autoimmunity is widely acknowledged to be impacted not only by genetic predisposition, but by environmental factors that potentially accelerate initiation of autoimmune responses and increase severity of organ-specific pathologies [1]. Systemic lupus erythematosus (SLE) is a prototypical autoimmune disease that affects 300,000 Americans, most of whom are women, and is often associated with glomerulonephritis and kidney failure [2]. In the United States, it is estimated that at least 1.7 million workers are employed in occupations (e.g. mining, construction, manufacturing and custodial industries) with potential exposure to crystalline silica (*c*SiO_2_) [3]. Human epidemiological studies have linked occupational exposure to crystalline silica to increased SLE incidence [4–6]. For example, a population-based, case-control study conducted with participants from the Carolina Lupus Cohort reported that development of SLE was more prevalent in individuals with occupations associated with *c*SiO_2_ exposure [5]. Similarly, in a cohort of 15,000 uranium miners heavily exposed to *c*SiO_2_, 28 men were diagnosed with SLE, whereas the predicted SLE prevalence for a group of this size was ≤ 1.5 individuals [7]. Finckh and coworkers [6] found that occupational exposure to *c*SiO_2_, but not organic solvents, contributed to increased SLE in urban women. Other studies report SLE-like symptoms in individuals with silicosis, including lymphocyte activation and elevated antinuclear antibodies, immunoglobulins, and immune complexes [8–11].

Both *in vivo* and *in vitro* studies have yielded several lines of evidence that *c*SiO_2_-induced cell death of alveolar macrophages is a critical event in *c*SiO_2_-induced exacerbation of autoimmunity [12–17]. Phagocytosis of *c*SiO_2_ particles by alveolar macrophages induces NF-κB activation [18,19]. This results in production of proinflammatory cytokines, including TNF-α [20], which are correlated with disease activity in individuals with SLE [21,22]. Aberrant apoptosis and/or necrosis of *c*SiO_2_-laden alveolar macrophages not only releases these particles, enabling continued pulmonary re-exposure to *c*SiO_2_ and sustained inflammation, but could also expose normally sequestered nuclear antigens (e.g. dsDNA). Both excessive apoptosis and defective clearance of apoptotic debris contribute to aberrant self-antigen presentation, driving consequent autoimmune sequealae [12,23].

Mouse strains that spontaneously develop SLE have been used to explore how toxicant exposure affects the pathogenesis of autoimmunity [24–27]. One such strain, NZM2410, bred from the widely used SLE model, NZBWF1 [24], exhibits rapid onset of lupus nephritis with most mice succumbing to glomerulonephritis by 25 wk of age [28]. When intranasally instilled with *c*SiO_2_, both male and female NZM2410 mice exhibit accelerated onset and increased severity of SLE-related responses including elevated autoreactive antibodies, proteinuria, and glomerulonephritis [29]. It was further determined that concentrations of TNF-α in bronchoalveolar lavage fluid of *c*SiO_2_-exposed NZM2410 mice were significantly higher than saline-treated control mice [30]. Autoantibodies isolated from sera of *c*SiO_2_-exposed NZM2410 mice were found to bind to apoptotic debris derived from alveolar macrophages [31]. Importantly, the effects of *c*SiO_2_ on autoimmunity could be attenuated in this strain *in vivo* by concurrent instillation with rottlerin, an inhibitor of apoptosis [32].

There are several issues that limit the use of the NZM2410 phenotype to uncover mechanisms by which *c*SiO_2_ triggers SLE. Not only does this strain develop rapid onset and progression of lupus nephritis compared to other lupus-prone mouse models [24], but the majority of NZM2410 mice succumb to glomerulonephritis within 2 wk of proteinuria appearance [33]. Furthermore, contrary to SLE prevalence in human population and other murine models [24], NZM2410 mice do not display female sex bias. Finally, while NZM2410 mice have been useful for delineating genetic contributions to SLE, most preclinical studies of putative therapeutic agents employ the NZBWF1 mouse, which displays slower development of autoimmunity [25]. Taken together, investigation of subtle underlying mechanisms and preventative measures to counter toxicant-triggered SLE might be enhanced by employing the less-penetrant NZBWF1 phenotype.

The purpose of this study was to test the hypothesis that short-term repeated intranasal *c*SiO_2_ exposure will both decrease latency to onset and increase severity of autoimmunity in female NZBWF1 mice. The results presented herein suggest that *c*SiO_2_ triggered robust autoimmune and inflammatory responses in the lung, including the formation of ectopic lymphoid tissue, that closely paralleled early onset of glomerulonephritis and exacerbated systemic autoimmune responses.

## Materials and Methods

### Mice

All experiments were approved by the Institutional Animal Care and Use Committee at Michigan State University in accordance with the National Institutes of Health guidelines for animal use. Female 7 wk old lupus-prone NZBWF1 and control C57Bl/6 mice were obtained from Jackson Laboratories (Bar Harbor, ME), randomized into treatment groups, and allowed to acclimate for 2 wk prior to *c*SiO_2_ exposure. Upon arrival and throughout the study, mice were fed semi-purified American Institute of Nutrition (AIN)-93G diet containing high oleic safflower oil [34]. Mice were housed 4 per cage with free access to food and water and maintained at constant temperature and humidity (21°C–24°C and 40–55%, respectively) under a 12-h light/dark cycle.

### *c*SiO_2_ exposure

**Fig 1:**
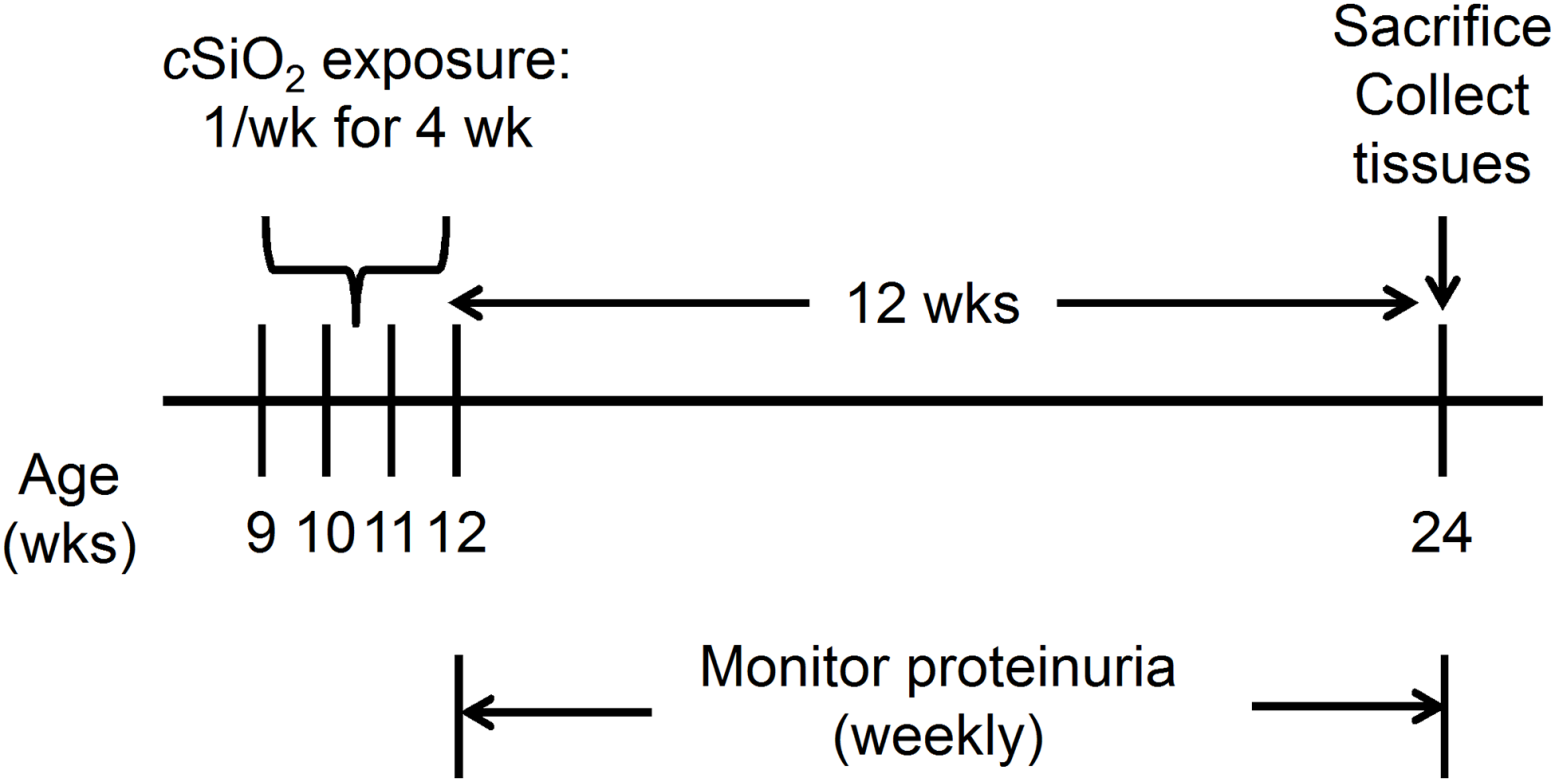
Experimental design for intranasal *c*SiO_2_ exposure. Beginning at 9 wk of age, NZBWF1 and C57Bl/6 mice were dosed intranasally with 25 μl PBS containing 0, 0.25 mg or 1.0 mg *c*SiO_2_ once per wk, for 4 wk. Proteinuria was monitored over the course of the experiment and all animals sacrificed at 24 wk of age.

The experimental design for *c*SiO_2_ exposure, summarized in Fig 1, was based on previous protocols [29–32,35]. *c*SiO_2_ (Min-U-Sil-5, average particle diameter 1.5–2.0 μm) was originally obtained from Pennsylvania Sand Glass Corporation (Pittsburgh, PA). Stock suspensions of each dose were prepared fresh in sterile phosphate buffered saline (PBS) just prior to intranasal exposure and sonnicated for 1 min before use. At 9 wk of age, mice were anesthetized with 4% isoflurane and instilled intranasally with 0, 0.25 and 1.0 mg of *c*SiO_2_ in 25 μl PBS. To mimic intense occupational exposure to *c*SiO_2_ over a short time period, mice were instilled once per wk for 4 wk. Urine was collected weekly and evaluated for proteinuria by 2P urine reagent strips (Cortez Diagnostics, Inc., Calabasas, CA). Mice were sacrificed and tissues collected 12 wk after the final exposure to *c*SiO_2_. At this time point, more than 50% of animals exposed to the high *c*SiO_2_ dose exhibited moderate proteinuria, defined as ≥ 300 mg/dl. In addition to NZBWF1, female C57Bl/6 mice were used as a control to discern histopathologic effects of high *c*SiO_2_ exposure in kidney and lung. C57Bl/6 mice were dosed with either 25 μl PBS or 1.0 mg *c*SiO_2_ in 25 μl PBS following the above protocol and terminated 12 wk after the final exposure.

### Necropsy and tissue collection

Animals were euthanized by intraperitoneal injection with 56 mg/kg BW sodium pentobarbital and exsanguination via the abdominal aorta. Blood was collected using heparinized syringes and centrifuged at 3500 x g for 10 min at 4°C for separation of plasma. The collected plasma was stored at -80°C until future analysis. Following BALF collection as described previously [36], lungs were intratracheally fixed with 10% (v/v) neutral buffered formalin at constant pressure (30 cm H_2_O) for a minimum 1 h and stored in fixative until further processing for histology. The right kidney was excised and the cranial portion fixed for 24 h in 10% neutral buffered formalin.

### Kidney histopathology

Formalin-fixed kidneys were paraffin-embedded, sectioned and stained with either hematoxylin and eosin (H&E) or Periodic acid-Schiff and hematoxylin (PASH). Stained sections were evaluated for lupus nephritis in a blinded fashion by a board-certified veterinary pathologist using a modified International Society of Nephrology/Renal Pathology (ISN/RPS) Lupus Nephritis Classification system [37]. Slide sections were graded as follows: (0) no tubular proteinosis and normal glomeruli; (1) mild tubular proteinosis with multifocal segmental proliferative glomerulonephritis and occasional early glomerular sclerosis and crescent formation; (2) moderate tubular proteinosis with diffuse segmental proliferative glomerulonephritis, early glomerular sclerosis and crescent formation; and (3) marked tubular proteinosis with diffuse global proliferative and sclerosing glomerulonephritis.

### Lung histopathology

Randomly orientated, serial sections of the formalin-fixed left lung lobe were processed routinely and embedded in paraffin. Tissue sections (5 μm) were deparaffinized and stained with H&E for histopathology. Tissues were semi-quantitatively scored by a board-certified veterinary pathologist for the following lung lesions: (a) presence of lymphocytic cell infiltration within perivascular and peribronchial regions, (b) alveolitis defined as the presence of alveolar infiltration of vacuolated macrophages, neutrophils, and lymphocytes, granuloma formation in the alveolus, type II epithelial cell hyperplasia, and thickened alveolar wall, and (c) presence of alveolar proteinosis. Individual lungs were graded for these lesions using the following criteria (% of total pulmonary tissue examined): (0) no changes compared to control mice; (1) minimal (<10%); (2) slight (10–25%); moderate (26–50%); (4) severe (51–75%); or (5) very severe (>75%) of total area affected.

Immunohistochemistry for IgG was performed on paraffin-embedded lungs. Tissue sections (5 μm) were deparaffinized and subjected to heat-induced epitope retrieval with citrate buffer (pH 6.0) for 30 min at 100°C. Endogenous peroxidase was blocked with 3% (v/v) H_2_O_2_/methanol for 30 min at room temperature followed by blocking non-specific protein binding with normal horse serum (Vector Laboratories Inc., Burlingame, CA) for 30 min. Tissue endogenous avidin and biotin binding sites were blocked by incubation with Avidin D (Vector Laboratories) followed by d-Biotin (Sigma Aldrich, St. Louis, MO), each for 15 min. Goat anti-mouse IgG γ-chain specific (Alpha Diagnostic Inc., San Antonio, TX) was diluted 1:4000 in normal antibody diluent (ScyTek Laboratories Inc., Logan, UT) and incubated for 60 min followed by incubation with 11μg/ml biotinylated horse anti-goat IgG H+L (Vector Laboratories, Inc.) in normal antibody diluent for 30 min. Peroxidase enzyme was incubated on slides for 30 min with Ready-to-Use Elite Peroxidase Reagent (Vector Laboratories, Inc.). Finally, slides were incubated for 15 min with Nova Red substrate (Vector Laboratories, Inc.) and counterstained with hematoxylin.

B and T lymphocytes were identified using rat anti-mouse CD45R (BD Biosciences, San Jose, CA) (1:300) and rabbit anti-mouse CD3 (1:100), respectively on deparaffinized tissue sections (4 μm) that were subjected to heat-induced epitope retrieval with citrate buffer (pH 6.0) for 30 min at 100°C (CD45R) or 125°C for 15 min (CD3). Following pretreatments, formation of avidin—biotin complex and primary antibody staining were performed as described above. Bound CD45R and CD3 antibodies were detected by incubation with biotinylated rat and goat anti-rabbit in conjunction with Peroxidase Reagent and Nova Red substrate.

### BALF processing and differential staining

Cytological slides from 150 μl BALF were prepared by centrifugation at 400 x g for 10 min using a Shandon Cytospin 3 (Shandon Scientific, Pittsburgh, PA), allowed to air dry, and stained with Diff-Quick (Fisher Scientific). A total of 200 cells were counted and cells identified as monocytes/macrophages, lymphocytes, and polymorphonuclear (PMN) leukocytes using morphological criteria. Remaining BALF was centrifuged at 2400 x g for 15 min and supernatant collected and stored at -80°C for IgG and cytokine analysis.

### IgG and autoantibody measurement

Total IgG, IgA and IgM were quantitated in BALF using an ELISA method described previously [38]. Briefly, 96-well plates were coated with 50μL of 10 μg/ml goat anti-mouse IgA, IgM, or IgG, respectively (Alpha Diagnostics, Inc.). The standard curve was generated using mouse reference serum (Bethyl Laboratories, Inc., Montgomery, TX). Plates were read on an ELISA reader (Molecular Devices, Menlo Park, CA) at 650 nm and Ig concentrations calculated from the standard curves using Softmax software (Molecular Devices). Total Ig antibodies for nuclear antigens and double-stranded DNA (dsDNA) in plasma were measured according to manufacturer’s instructions using commercial ELISA kits (Alpha Diagnostics, Inc.).

### Cytokine analysis

BALF and plasma were diluted two-fold and analyzed for the inflammatory cytokines IL-6, IL-10, MCP-1, IFN-γ, TNF, and IL-12p70 using a Mouse Inflammation Cytometric Bead Array (BD Biosciences). Data was acquired using a FACSCalibur flow cytometer (BD Biosciences) and cytokine concentrations were calculated from standard curves using FCAP Array Software (BD Biosciences).

### Statistics

All treatment groups consisted of 8 mice and data presented as group mean ± SEM. Data were plotted and analyzed using SigmaPlot 11.0 for Windows (Jandel Scientific; San Rafael, CA). Grubb’s test was performed to identify and exclude outliers from statistical analysis. Differences between *c*SiO_2_- and vehicle-treated NZBWF1 mice were analyzed by one-way ANOVA on Ranks with Dunn’s method. Differences between *c*SiO_2_- and vehicle-treated C57Bl/6 mice, and NZBWF1 and C57Bl/6 histopathological lung grades were analyzed by Mann-Whitney Rank Sum Test. The Spearman rank-order correlation coefficient was used to correlate *c*SiO_2_ dose to experimental endpoint for NZBWF1. A P value of < 0.05 was considered statistically different for all study outcomes.

## Results

### *c*SiO_2_ elicits early onset of proteinuria and glomerulonephritis

**Fig 2:**
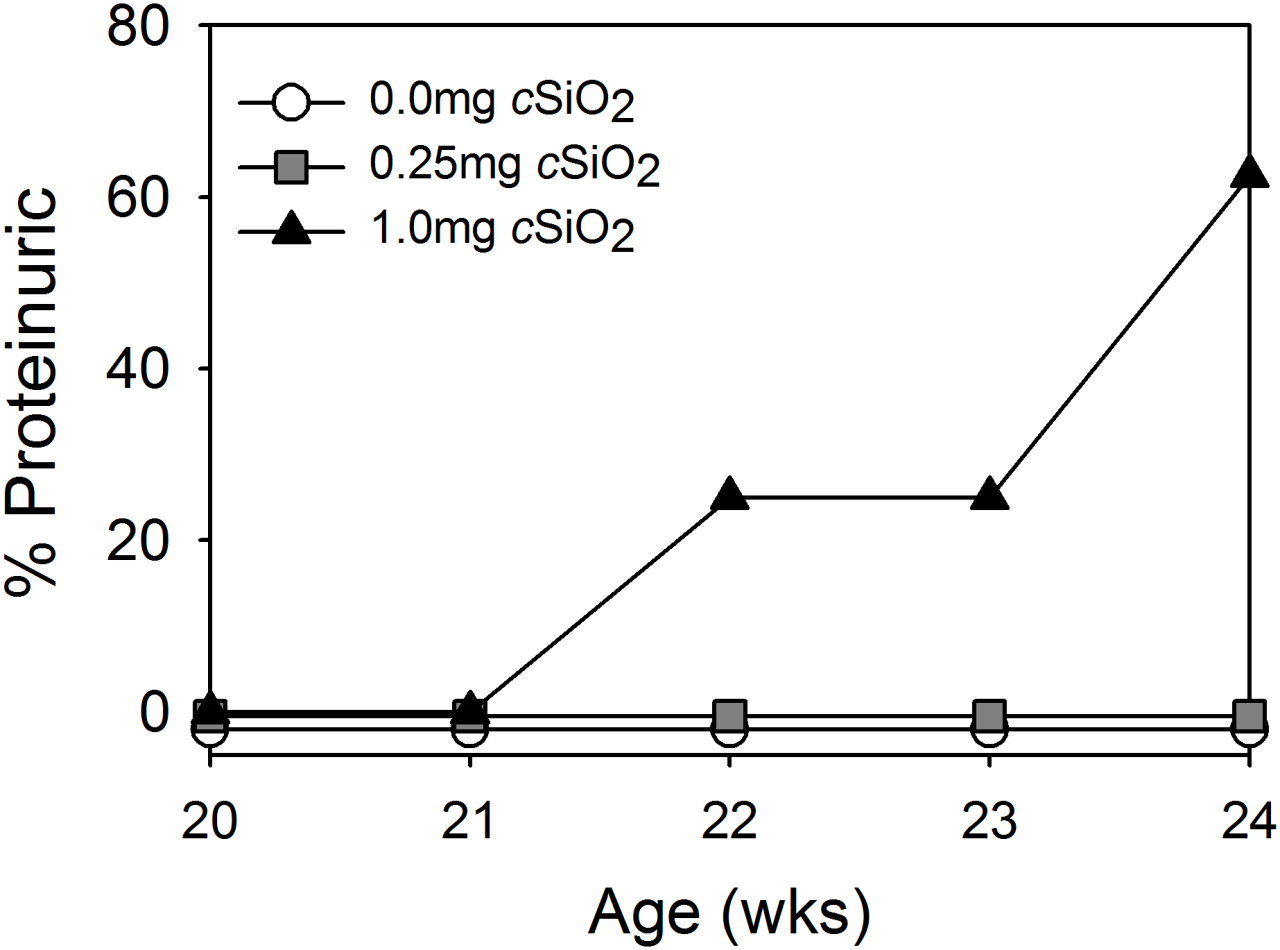
*c*SiO_2_ exposure accelerates development of proteinuria in NZBWF1 mice. Proteinuria was monitored weekly until sacrifice 12 wk after the final *c*SiO_2_ exposure when most mice exposed to 1.0 mg *c*SiO_2_ were over threshold (≥ 300 mg/dl). Proteinuria was not detected in NZBWF1 mice dosed with 0.25 mg *c*SiO_2_ or vehicle. C57Bl/6 mice exposed to *c*SiO_2_ or vehicle did not develop detectable proteinuria over the course of the experiment.

The effects of weekly intranasal exposure to 0, 0.25 mg, or 1.0 mg cSiO_2_ from 9 to 12 wk of age on renal function were monitored by measuring urinary protein at weekly intervals. Proteinuria (≥ 300 mg/dl) was observed in 13% and 62% of NZBWF1 mice treated with 1.0 mg *c*SiO_2_ at 10 and 12 wk post-dosing, respectively (Fig 2). In contrast, proteinuria was not evident in NZBWF1 mice dosed with 0.25 mg *c*SiO_2_ or vehicle up to 12 wk post-treatment. Proteinuria was not observed in control C57Bl/6 mice treated with vehicle or 1.0 mg *c*SiO_2_ up to 12 wk post-dosing.

**Fig 3:**
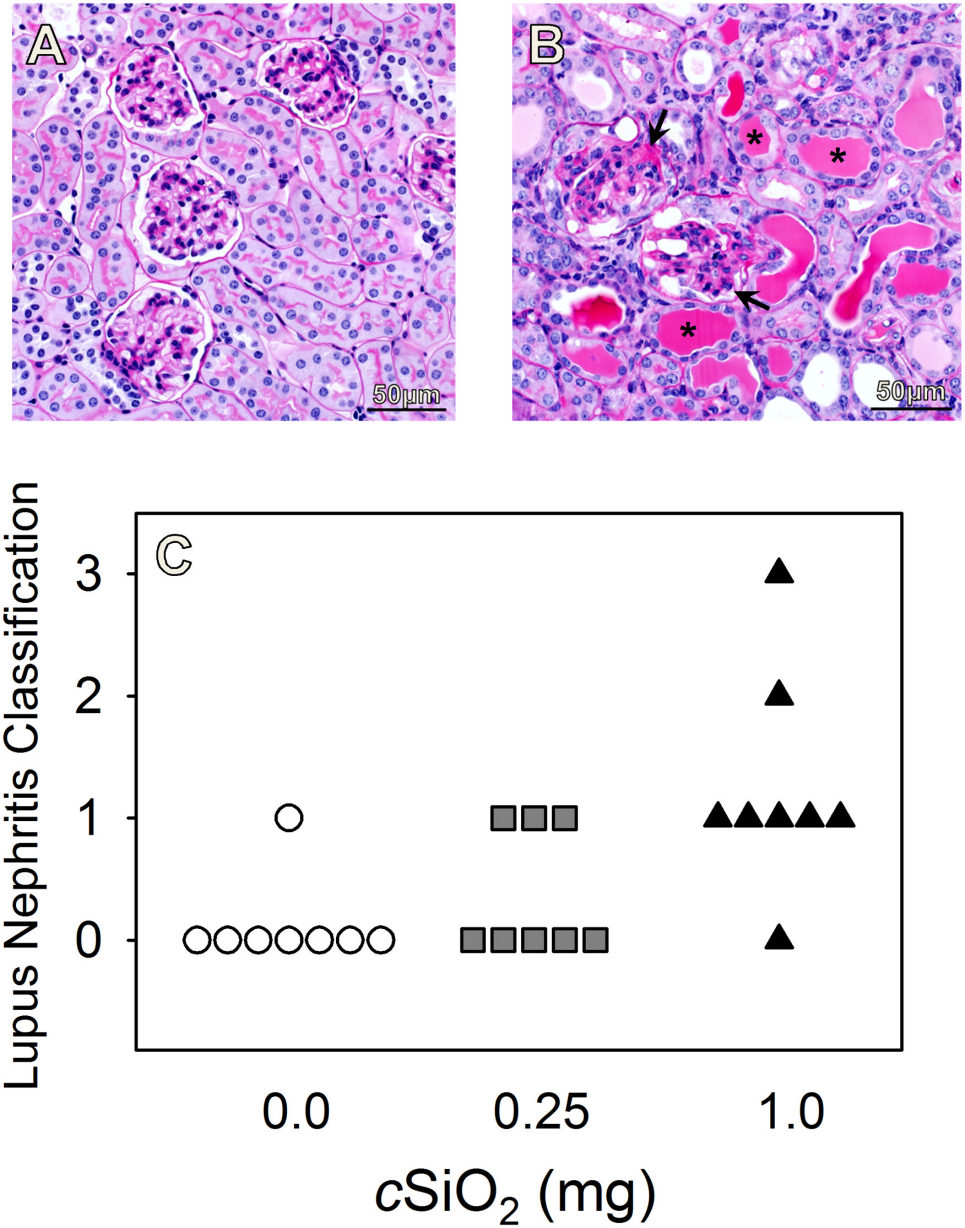
*c*SiO_2_ exposure increases severity of lupus nephritis in kidneys of NZBWF1 mice. Representative light photomicrographs of PASH stained kidney sections in NZBWF1 mice at 24 wks of age exposed to vehicle (A) and 1.0mg *c*SiO_2_ (B). NZBWF1 mice exposed to 1.0mg *c*SiO_2_ developed extensive glomerulonephritis (black arrow) and tubular proteinosis (asterisk). NZBWF1 mice were individually graded for lupus nephritis following the modified ISN/RPS classification system as described in the Materials and Methods (C). Animals exposed to *c*SiO_2_ developed more severe lesions characteristic of lupus nephritis than vehicle-exposed mice. *c*SiO_2_ dose significantly correlated with lupus nephritis (Spearman rank-order coefficient = 0.64, *p* < 0.05).

**Fig 4:**
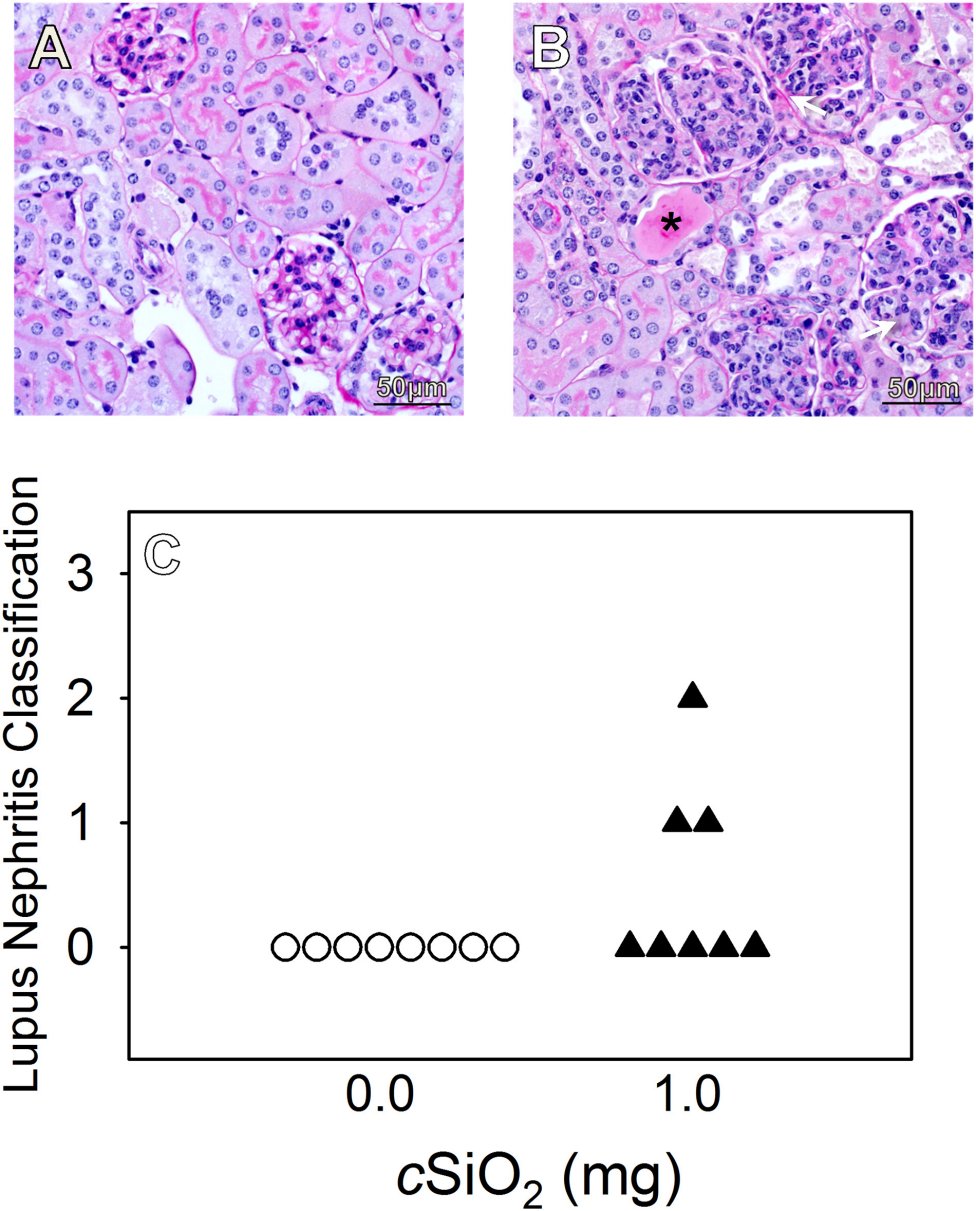
*c*SiO_2_ exposure in C57Bl/6 mice induced kidney lesions resembling lupus nephritis. Representative light photomicrographs of PASH stained kidney sections in C57Bl/6 mice at 24 wks of age exposed to vehicle (A) and 1.0mg *c*SiO_2_ (B). Some renal histopathological lesions were observed in *c*SiO_2_-exposed C57Bl/6 mice (B) with notable renal tubular proteinosis (asterisk) and several glomeruli with global mesangial hypercellularity (white arrows). C57Bl/6 mice were individually graded for lupus nephritis as described in the Materials and Methods (C). Animals exposed to *c*SiO_2_ developed more severe lesions characteristic of lupus nephritis than vehicle exposed mice.

To confirm nephritogenic effects of *c*SiO_2_, kidney sections from NZBWF1 mice at 12 wk post-treatment were assessed histopathologically following H&E and PASH staining (Fig 3A and 3B). Kidneys from NZBWF1 mice treated with 1.0 mg *c*SiO_2_ exhibited frequent tubular proteinosis and proliferative glomerulonephritis (Fig 3B). Lymphocytic infiltration at the renal pelvis as well as sclerosis and crescent formation were additional common findings in animals treated at this dose. These responses were largely absent in NZBWF1 mice treated with vehicle (Fig 3A) or 0.25 mg *c*SiO_2_ as well as control C57Bl/6 mice treated with vehicle or 1.0 mg *c*SiO_2_ (Fig 4A and 4B).

When mice were graded individually for glomerulonephritis severity, only 13% (1/8) of NZBWF1 mice treated with vehicle were ranked 1 due to the occurrence of minimal tubular proteinosis and rare glomeruli with segmental mesangial hypercellularity, whereas the remaining vehicle-treated mice were ranked 0 (Fig 3C). In contrast, *c*SiO_2_ elicited renal lesions characteristic of lupus nephritis, with 38% (3/8) and 63% (5/8) of NZBWF1 mice treated with 0.25 mg and 1.0 mg of the agent, respectively, being ranked 1. Additionally, 25% (2/8) of NZBWF1 mice treated with 1.0 mg *c*SiO_2_ had renal lesions ranked 2 or 3, whereas no mice treated with vehicle or 0.25 mg *c*SiO_2_ developed such severe lesions. Interestingly, while all C57Bl/6 mice exposed to vehicle were ranked 0, indicating no renal lesions consistent with glomerulonephritis, 38% of mice dosed with 1.0 mg *c*SiO_2_ developed renal lesions ranked 1 or 2 (Fig 4C). Thus, *c*SiO_2_ appeared to induce modest glomerulonephritis in some of the C57Bl/6 control mice without onset of evident proteinuria.

### *c*SiO_2_ induces marked lymphoplasmacytic infiltration in lung parenchyma

**Fig 5:**
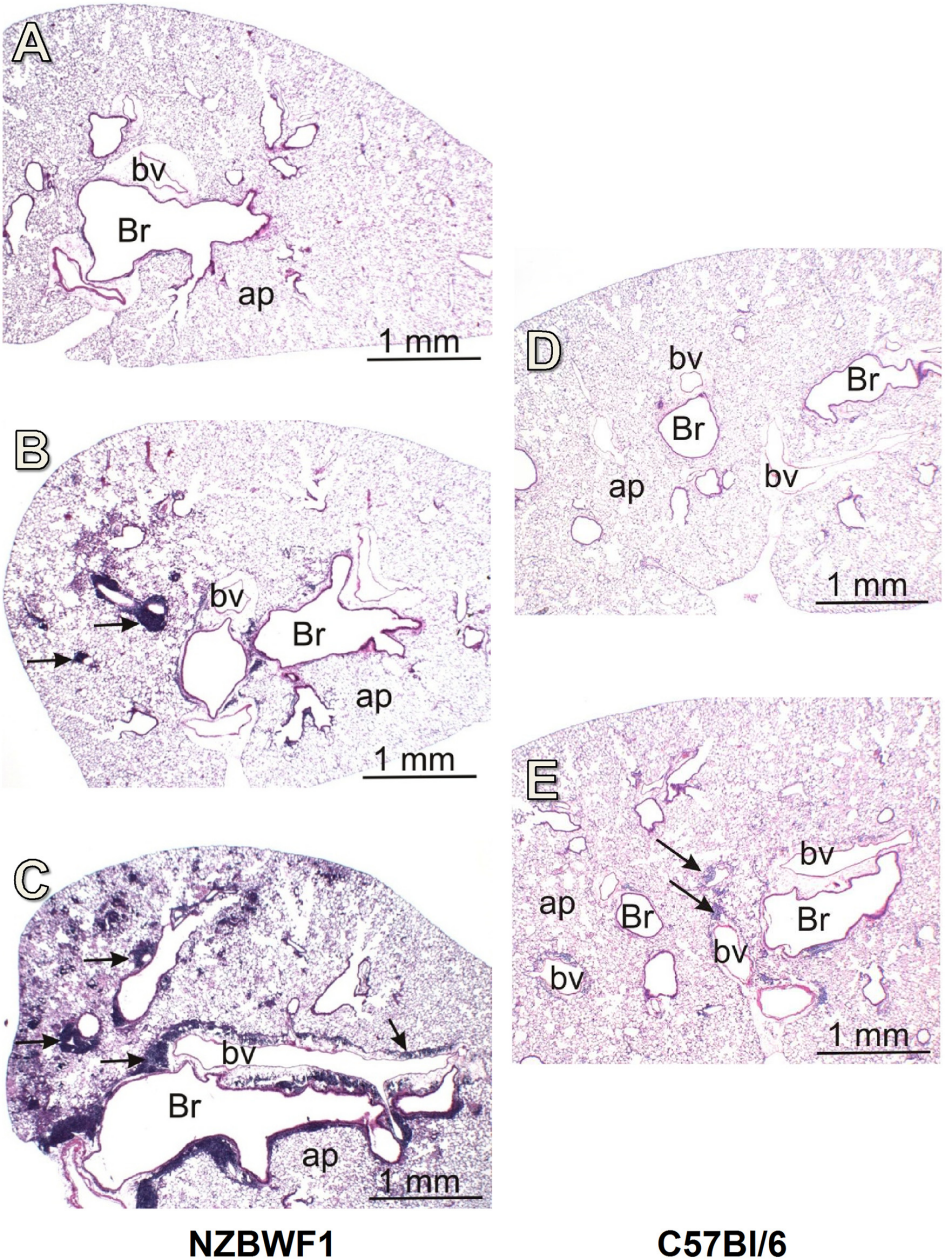
*c*SiO_2_ elicits perivascular and peribronchiolar lymphocytic infiltration in lungs of NZBWF1 and C57Bl/6 mice. Representative light photomicrographs of H&E stained lung sections from NZBWF1 mice exposed to vehicle (A), 0.25 mg *c*SiO_2_ (B), or 1.0 mg *c*SiO_2_ (C) and C57Bl/6 mice given vehicle (D), or 1.0mg *c*SiO_2_ (E). Br = bronchiole, bv = bronchial vasculature, ap = alveolar parenchyma. Black arrows indicate lymphocytic infiltration in perivascular and peribronchial regions. Lymphocytic infiltration was semi-quantitatively graded as described in the Materials and Methods (Table 1).

Histologically, conspicuous lymphoplasmacytic infiltrates were present in interstitial tissues surrounding bronchioles and associated blood vessels throughout the lung in NZBWF1 mice exposed to 1.0 mg *c*SiO_2_ (Fig 5C). This response was less severe in NZBWF1 mice treated with 0.25 mg *c*SiO_2_ and minimal in NZBWF1 mice treated with vehicle (Fig 5A and 5B; Table 1). C57Bl/6 mice exposed to 1.0 mg *c*SiO_2_ had minimal perivascular and peribronchial lymphoplasmacytic infiltrates as compared to vehicle controls (Fig 5D and 5E) and NZBWF1 mice treated with *c*SiO_2_ (Table 1).

**Table 1:** *c*SiO_2_ exposure increases severity of lymphocytic cell infiltration in lungs of NZBWF1 mice relative to C57Bl/6 mice.

Histopathological Lesion	*c*SiO_2_ dose				
	NZBWF1			C57Bl/6	
	0.0 mg	0.25 mg	1.0 mg	0.0 mg	1.0 mg
Lymphocytic cell infiltration	0.8 ± 0.2	2.5 ± 0.3*	3.4 ± 0.2*^, b^	0.0 ± 0.0	2.3 ± 0.2*^, a^
Alveolitis	0.0 ± 0.0	1.5 ± 0.2*	2.5 ± 0.3*^, a^	0.0 ± 0.0	2.3 ± 0.3*^, a^
Alveolar proteinosis	0.0 ± 0.0	1.4 ± 0.2*	2.6 ± 0.2*^, a^	0.0 ± 0.0	2.6 ± 0.2*^, a^

Mice were graded individually for severity of lung inflammation (% of total pulmonary tissue examined) as follows: 0, no changes; 1, minimal (<10%); 2, slight (10–25%); 3, moderate (26–50%); 4, severe (51–75%) 5; very severe (>75%) of total area affected. Data are mean ± SEM (n = 8/gp). NZBWF1 mice were analyzed by One-way ANOVA on Ranks followed by Spearman rank-order correlation. C57Bl/6 mice were analyzed by Mann-Whitney Rank Sum Test. Comparisons between strains at 1.0mg *c*SiO_2_ were analyzed for statistical differences by Mann-Whitney Rank Sum Test. Asterisks indicate statistical difference between silica treatment and vehicle control (*p* < 0.05). Different letters indicate statistical difference in histopathological lesions between strains (*p* < 0.05). *c*SiO_2_ dose in NZBWF1 significantly correlated (*p* < 0.05) with lymphocytic cell infiltration (Spearman rank-order correlation coefficient = 0.87), alveolitis (Spearman rank-order correlation coefficient = 0.90), and alveolar proteinosis (Spearman rank-order correlation coefficient = 0.94)

In addition to the pronounced inflammatory cell infiltration observed in the interstitial tissue of the lung, diffuse alveolar lesions were histologically evident after *c*SiO_2_ exposure in NZBWF1 and C57Bl/6 mice. In contrast to the aforementioned lymphocytic response in the lung tissues, the severity of alveolar lesions in NZBWF1 mice was comparable to C57Bl/6 mice given the same amount of *c*SiO_2_ (Table 1). Dosing of NZBWF1 mice with 0.25 mg *c*SiO_2_ also elicited the responses observed with 1.0 mg *c*SiO_2_ in this strain, albeit to a more modest extent.

### *c*SiO_2_ elicits IgG producing plasma cells in lung

**Fig 6:**
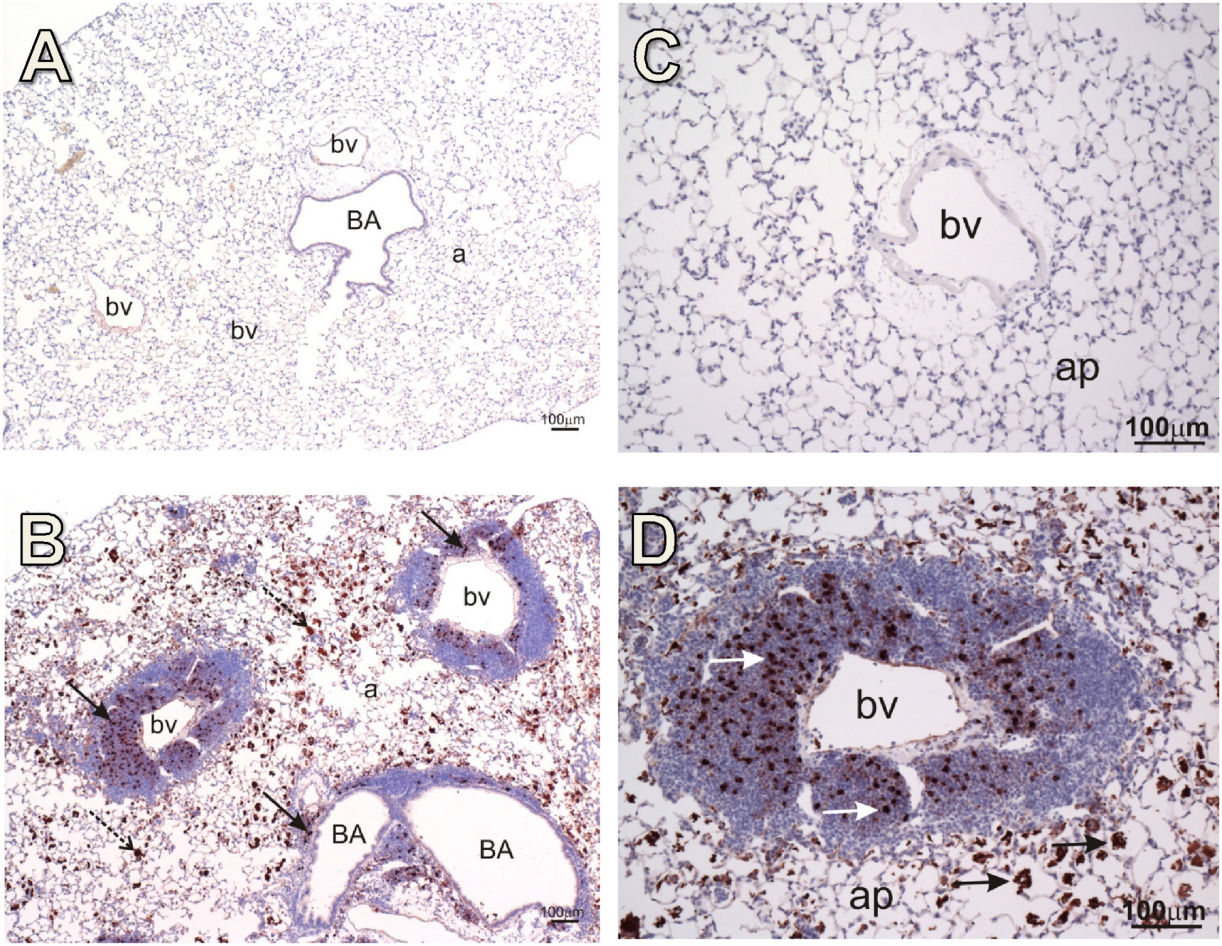
Marked accumulation of IgG producing plasma cells occurs in lungs of *c*SiO_2_-exposed NZBWF1 mice. Representative immunohistochemical photomicrographs of IgG in lungs of NZBWF1 mice. Photomicrographs taken at low magnification are shown in A and B whereas images at high magnification are shown in C and D. Vehicle-exposed mice did not indicate positive IgG staining (A,C). *c*SiO_2_ induced marked infiltration of IgG-laden lymphocytes peripheral to both blood vessels (bv) and bronchiole airways (BA) (black arrows in B, white arrows in D). IgG was also detected extracellularly within alveolar parenchyma (ap) (stippled arrows in B, black arrows in D).

Based on the prominent lymphoplasmacytic response in the lungs of *c*SiO_2_-treated NZBWF1 mice, the effects of intranasal *c*SiO_2_ on IgG production in these tissues was assessed immunohistochemically. Numerous IgG-laden lymphocytes were found present in the perivascular and peribronchiolar lymphoplasmacytic aggregates in *c*SiO_2_-treated but not vehicle-treated NZBWF1 mice (Fig 6B and 6D). In addition, H&E staining of lung sections from NZBWF1 mice treated with *c*SiO_2_ revealed occasional plasma cells containing Russell bodies (conspicuous cytoplasmic accumulation of hyaline material) that were immunohistochemically identified as containing IgG globulin. Taken together, these results suggest that both production and secretion of IgG by infiltrating plasma cells is induced in both lungs and airways after intranasal *c*SiO_2_ exposure in NZBWF1 mice.

### CD45R+ and CD3+ infiltrating lymphocytes in lung organize into ectopic lymphoid tissue after *c*SiO_2_ exposure in NZBWF1 mice

**Fig 7:**
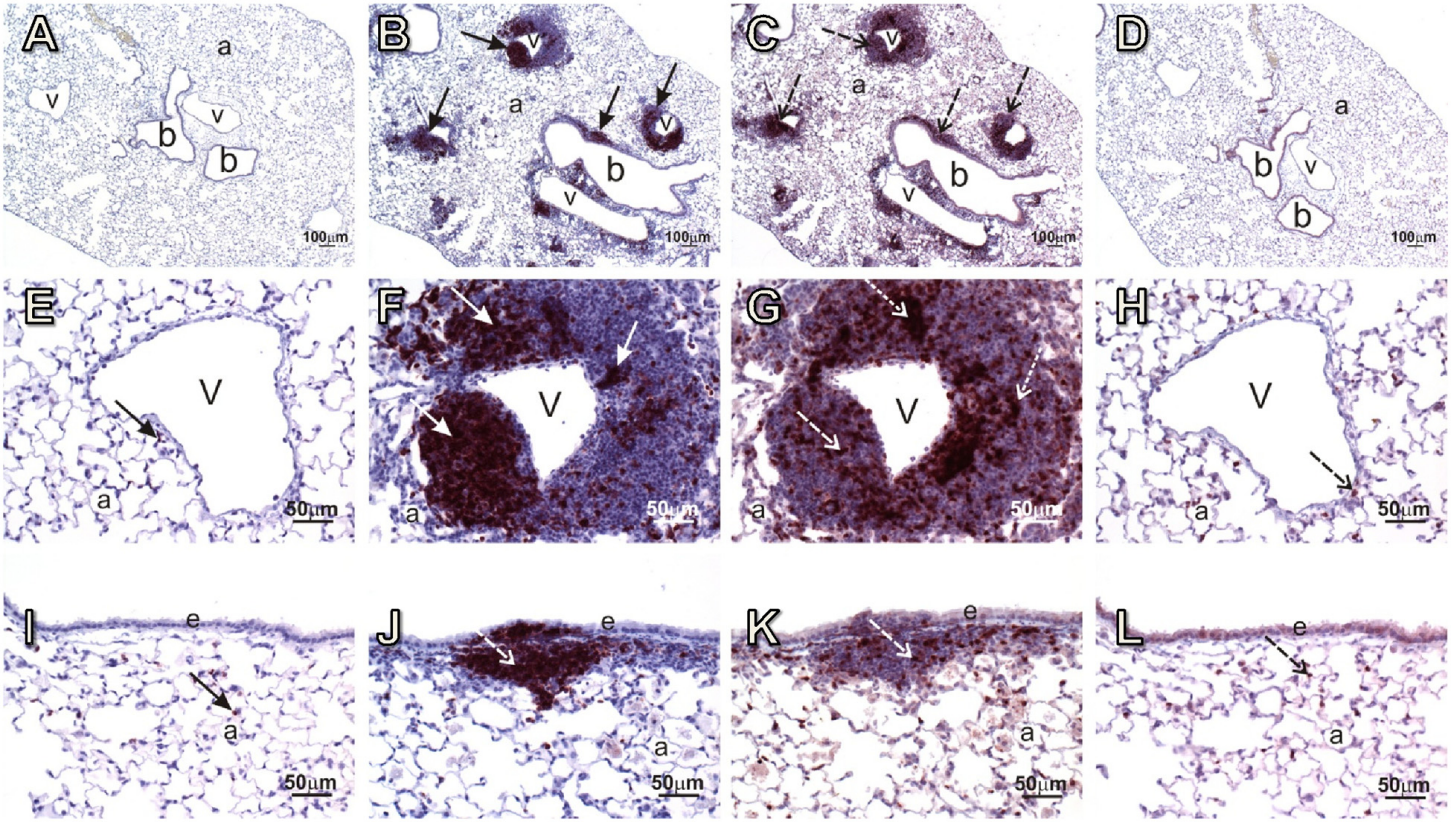
Intranasal *c*SiO_2_ exposure induces infiltration of CD45R+ and CD3+ lymphocytes in lungs that resemble ectopic lymphoid tissue. Representative light photomicrographs of lung tissue sections from mice treated with 0.0 mg *c*SiO_2_ (vehicle controls; A, D, E, H, I and L) or 1.0 mg *c*SiO_2_ (B, C, F, G, J and K). Some lung sections were immunohistochemically stained to identify B lymphocytes (CD45R+) (A, B, E, F, I, and J), while others (C, D, G, H, K, and L) were immunohistochemically stained to identify T lymphocytes (CD3+). Photomicrographs taken at high magnifications of blood vessels (v) and bronchioles (b) are illustrated in E, F, G, H and I, J, K, L, respectively. In *c*SiO_2_-treated mice, both bronchiolar airways and blood vessels were fully or partially circumscribed by thick interstitial infiltrates of mononuclear cells (arrows in B and C). These infiltrates were primarily comprised of B lymphocytes (solid arrows in F and J) and T lymphocytes (stippled arrows in G and K). B cells tended to form distinct focal aggregates (solid arrows in F and J) and T cells (stippled arrows in G and K) were more diffusely distributed throughout the peribronchiolar and perivascular lymphoid infiltrates. Control mice had only a few widely scattered B (solid arrow in E, I) and T (stippled arrow in H, L) cells present in the alveolar parenchyma (a), but no distinct lymphoid cell cuffing around the bronchioles or blood vessels. All tissues were counterstained with hematoxylin. e, airway epithelium.

Infiltrating lymphocytes induced by *c*SiO_2_ in lungs of NZBWF1 mice were further characterized by immunohistochemistry for B and T lymphocytes. Distinct aggregates of CD45R+ cells (B lymphocytes) (Fig 7B, 7F and 7J) interspersed with CD3+ cells (T lymphocytes) (Fig 7C, 7G and 7K) were observed peripheral to both bronchiolar airways and blood vessels in lung interstitial tissue of NZBWF1 mice exposed to 1.0 mg *c*SiO_2_. The distinct localization of CD45R+ aggregated cells in conjunction with infiltrating CD3+ lymphocytes in the lungs following *c*SiO_2_ exposure in NZBWF1 mice structurally resembled the spatial architecture of ectopic lymphoid tissue (ELT). The identification of ELT in parallel with the observation of IgG-producing plasma cells suggest that these structures functionally contribute to initiation of antigen-specific adaptive immune responses in lungs of NZBWF1 mice following intranasal *c*SiO_2_ exposure.

### cSiO_2_ induces leukocyte infiltration and elevates IgG, IgA, and IgM in BALF

**Fig 8:**
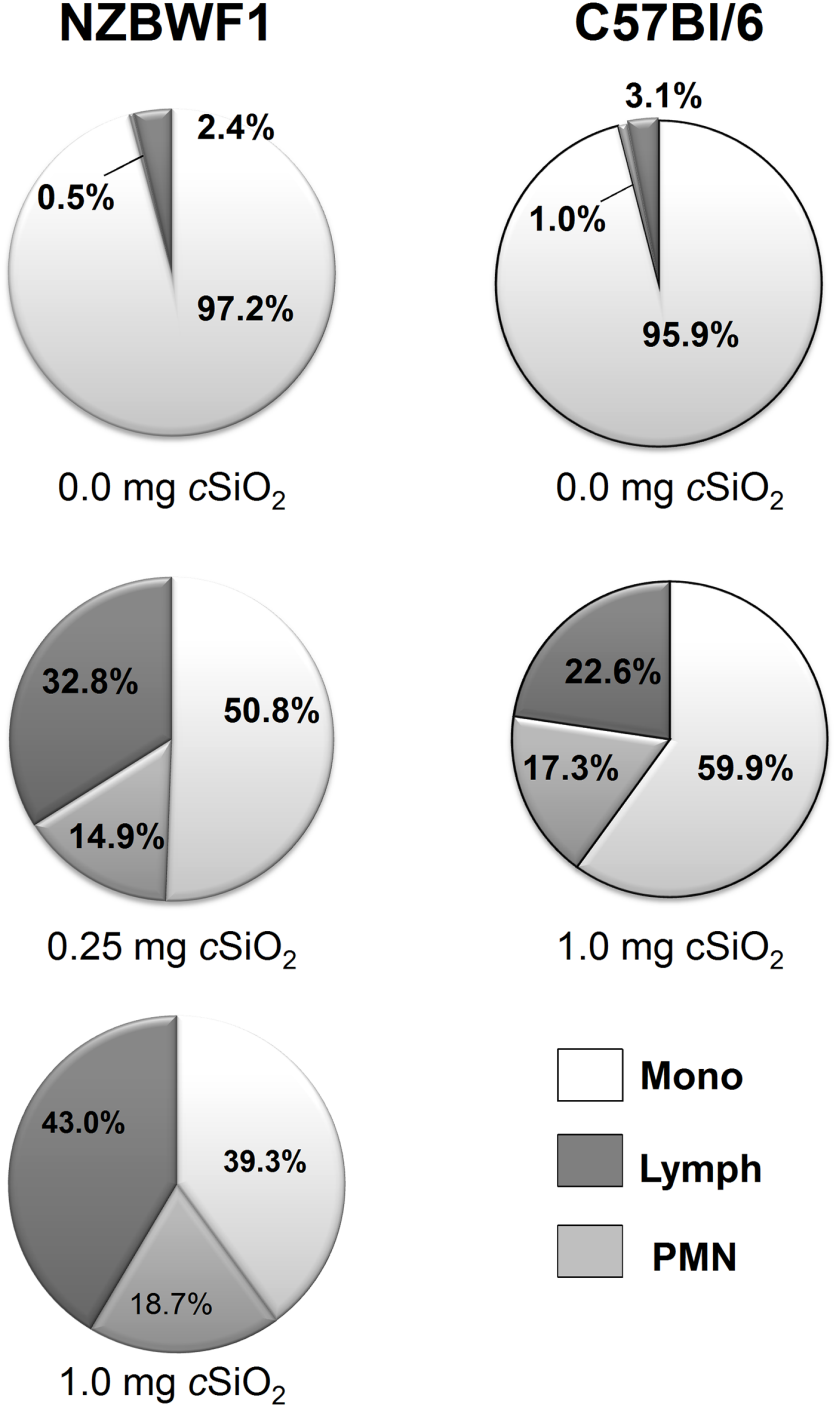
Intranasal *c*SiO_2_ exposure induces infiltration of lymphocytes and PMN leukocytes in BALF of NZBWF1 and C57Bl/6 mice. Cytometric slides prepared from BALF were stained with Diff-Quick and 200 cells per slide identified as monocytes/macrophages (Mo), lymphocytes (Lymph) or polymorphonuclear (PMN) cells. In NZBWF1 mice, group mean ± SEM at 0.0 mg *c*SiO_2_ were 97.2 ± 0.4%, 2.4 ± 0.3%, and 0.5 ± 0.1% for Mo, Lymph and PMN, respectively. Group mean ± SEM for 0.25 mg *c*SiO_2_ group were 50.8 ± 2.9%, 32.8 ± 3.5%, and 14.0 ± 1.8%, respectively and for the 1.0 mg *c*SiO_2_ group were 39.3 ± 1.7%, 43.0 ± 2.5%, and 18.7 ± 1.8%, respectively. In C57Bl/6 mice, group mean ± SEM at 0.0 mg *c*SiO_2_ were 95.9 ± 0.9%, 3.1 ± 0.7% and 1.0 ± 0.2% for Mo, Lymph, and PMN, respectively. Group mean ± SEM for 1.0mg *c*SiO_2_ were 59.9 ± 3.7%, 22.6 ± 3.7, and 17.3 ± 2.1% for Mo, Lymph, and PMN, respectively.

Effects of enhanced lymphocytic infiltration following intranasal *c*SiO_2_ exposure were also evident in the airways. In the recovered BALF, treatment with 0.25 mg and 1.0 mg *c*SiO_2_ in NZBWF1 mice elicited increased recruitment of lymphocytes (33% and 43%, respectively) relative to vehicle control where the majority of cells were monocytes/macrophages in both NZBWF1 and C57Bl/6 mice (Fig 8). Relative to NZBWF1 mice, 1.0 mg *c*SiO_2_ in C57Bl/6 mice elicited a modest increase of lymphocytes in BALF (23%). Similar PMN leukocyte responses were observed after 1.0mg *c*SiO_2_ exposure in both NZBWF1 (19%) and C57Bl/6 mice (17%).

**Fig 9:**
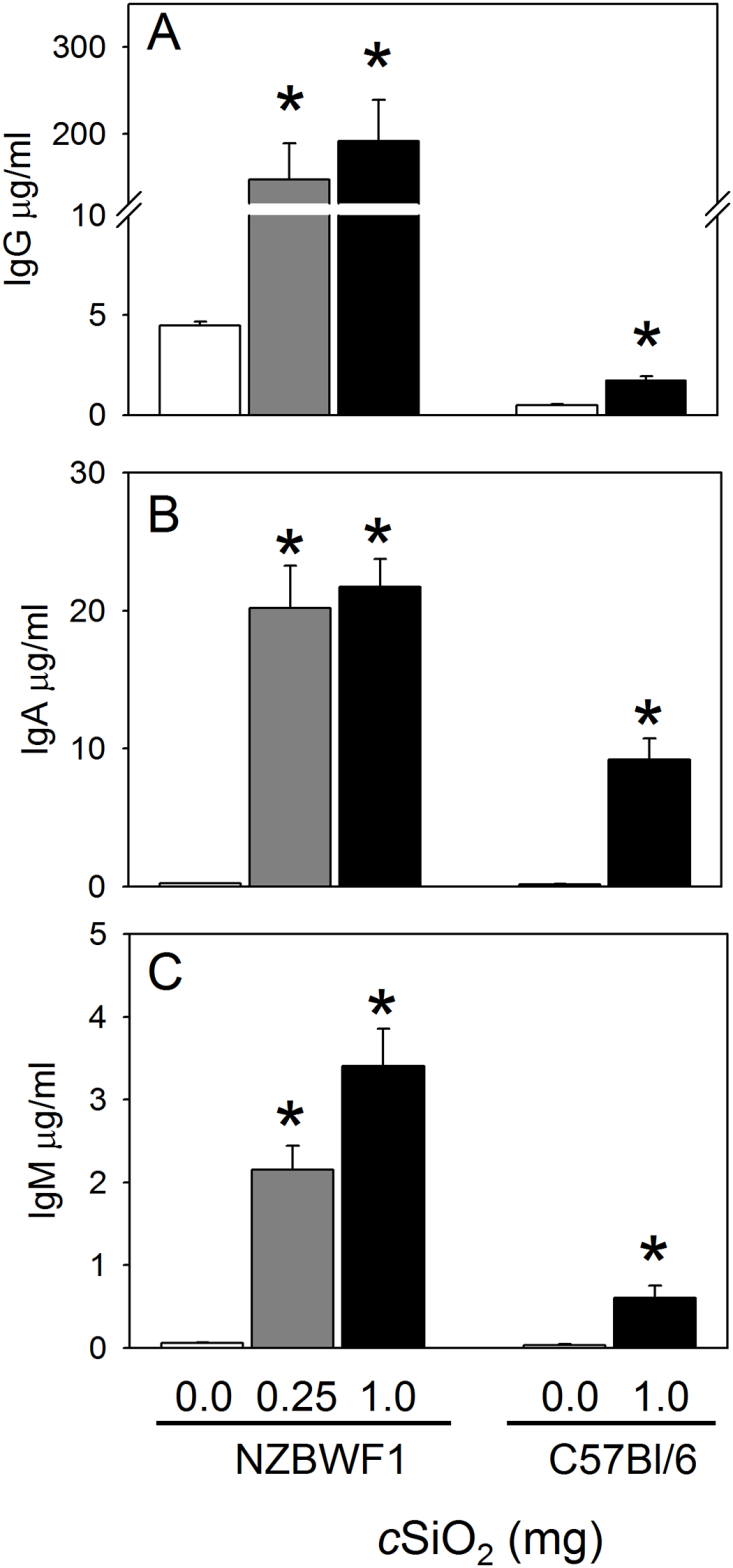
*c*SiO_2_ exposure elevates IgG (A), IgA (B), and IgM (C) concentrations in BALF of NZBWF1 and C57Bl/6 mice. Total immunoglobulins in BALF were measured by ELISA. Data are group mean ± SEM (n = 7–8/gp) and were analyzed by one-way ANOVA on Ranks with Dunn’s method (NZBWF1) or Mann-Whitney Rank Sum Test (C57Bl/6). Asterisk indicates a statistically significant difference between *c*SiO_2_ treatment and vehicle control (*p* < 0.05). In BALF of NZBWF1 mice, *c*SiO_2_ dose correlated significantly (*p* < 0.05) with IgG (Spearman rank-order correlation coefficient = 0.80), IgA (Spearman rank-order correlation coefficient = 0.72), and IgM (Spearman rank-order correlation coefficient = 0.85).

Based on the observed increase in lymphocytes following intranasal *c*SiO_2_ exposure, we performed ELISA to quantitate IgG, IgA and IgM in BALF of NZBWF1 and C57Bl/6 mice. IgG in BALF of NZBWF1 was substantially elevated in a dose-dependent manner following *c*SiO_2_ treatment (Fig 9A). *c*SiO_2_ also increased IgA and IgM in BALF of both NZBWF1 and C57Bl/6 mice, albeit to a lesser extent than IgG (Fig 9B and 9C).

### *c*SiO_2_ induces elevation of MCP-1, TNF-α, and IL-6 in BALF

**Fig 10:**
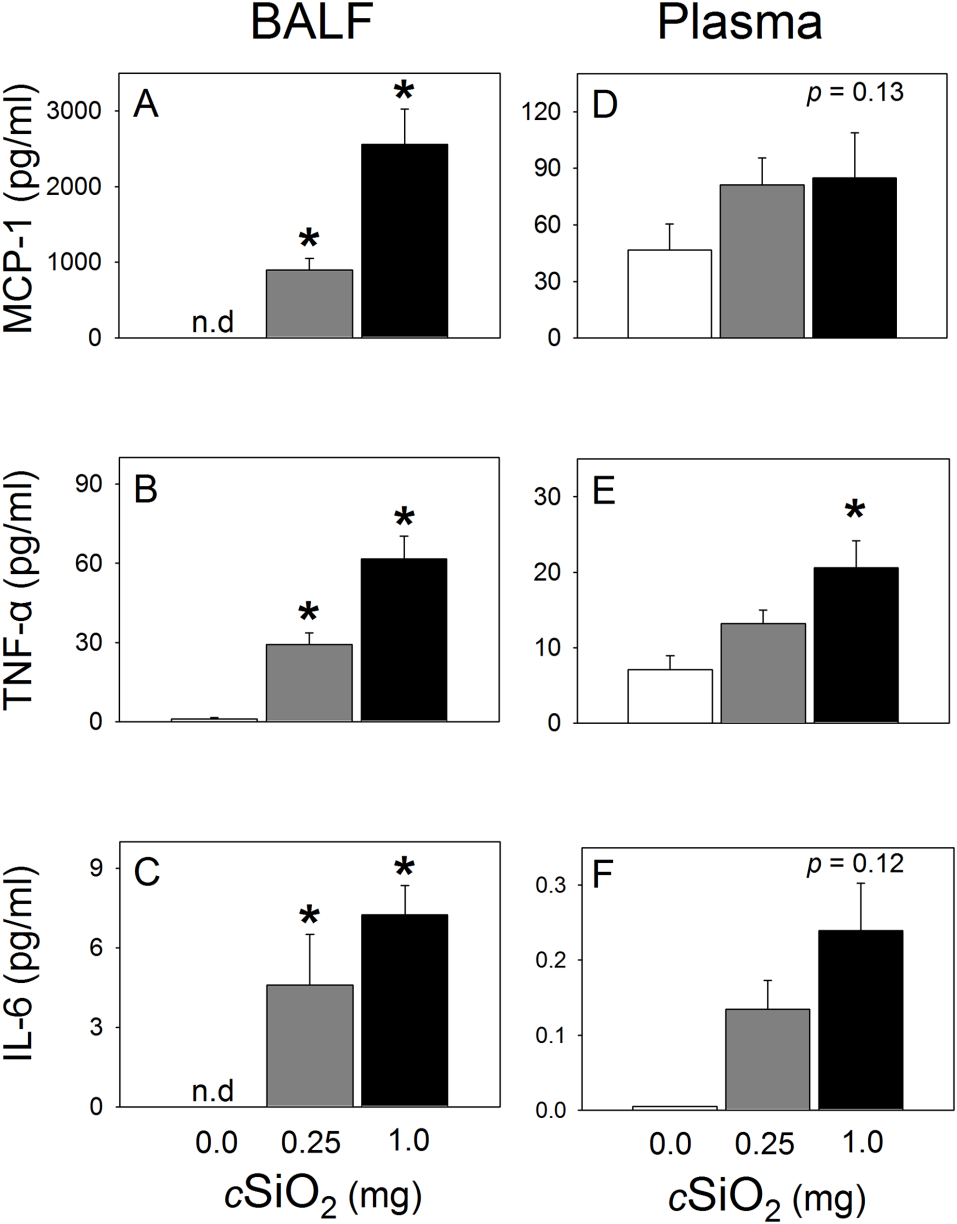
*c*SiO_2_ exposure increases proinflammatory cytokines MCP-1 (A, D), TNF-α (B, E), and IL-6 I (C, F) in BALF and plasma of NZBWF1 mice. Proinflammatory cytokine concentrations in BALF and plasma were determined by cytometric bead array. Data are group mean ± SEM (n = 5–8/gp) and were analyzed by one-way ANOVA on Ranks with Dunn’s method. Asterisk indicates a statistically significant difference in analyte between *c*SiO_2_ treatment and vehicle control (*p* < 0.05). *c*SiO_2_ dose correlated significantly (*p* < 0.05) with BALF MCP-1 (Spearman rank-order correlation coefficient = 0.90), BALF TNF-α (Spearman rank-order correlation coefficient = 0.89), and BALF IL-6 (Spearman rank-order correlation coefficient = 0.82). *c*SiO_2_ dose also correlated significantly (*p* < 0.05) with plasma TNF-α (Spearman rank-order correlation coefficient = 0.60), and IL-6 (Spearman rank-order correlation coefficient = 0.49). n.d. indicates not detected.

BALF of NZBWF1 mice was further analyzed for the cytokines IL-6, IL-10, MCP-1, IFN-γ, TNF-α, and IL-12p70. While effects on IFN-γ, IL-10 or IL-12p70 were not detectable, *c*SiO_2_ treatment dose-dependently induced elevation of the proinflammatory cytokines MCP-1, TNF-α, and IL-6 (Fig 10A–10C).

### *c*SiO_2_ triggers systemic autoantibody and proinflammatory cytokine responses

**Fig 11:**
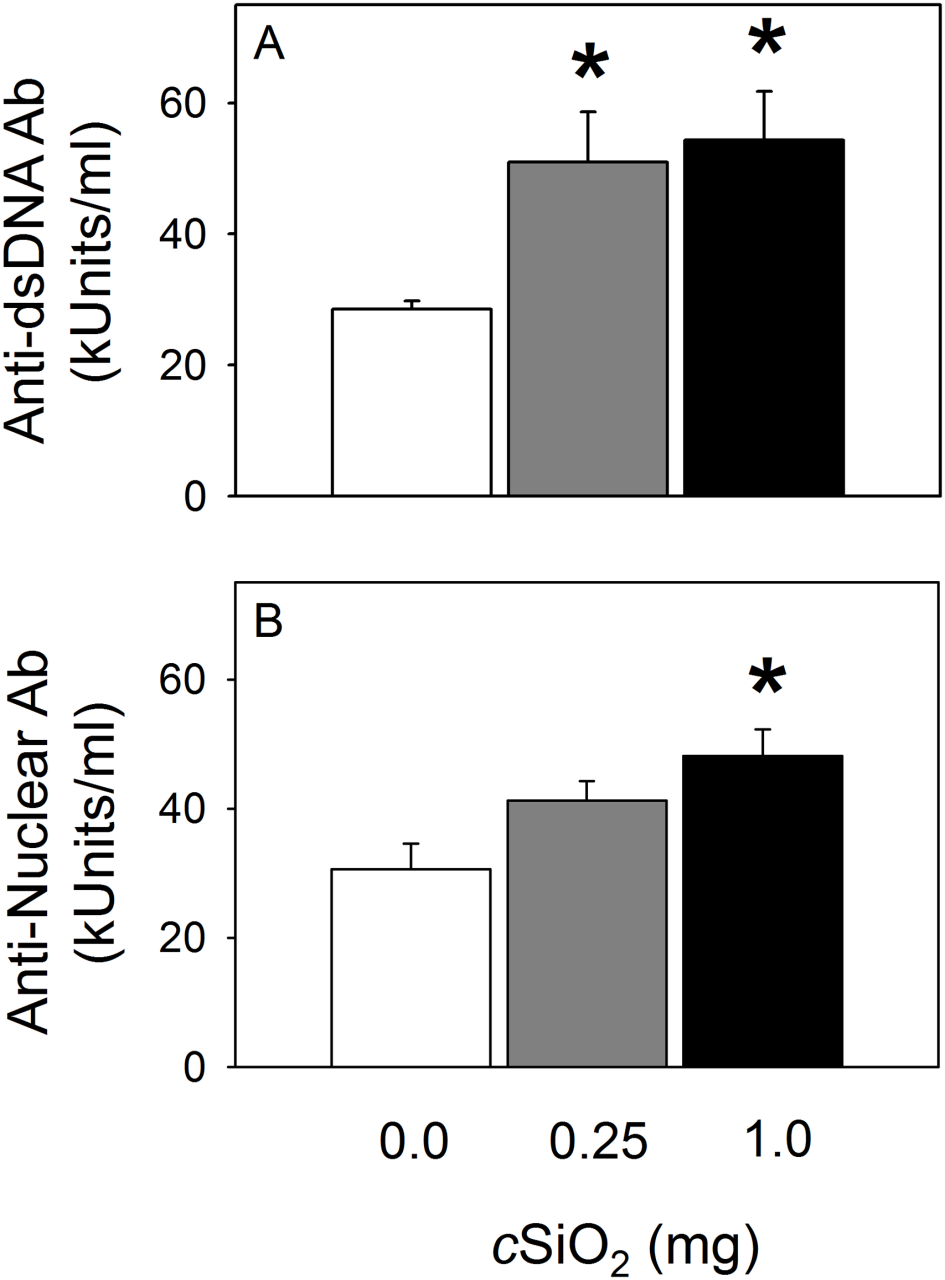
*c*SiO_2_ exposure increases anti-dsDNA antibodies (A) and anti-nuclear antibodies (B) in plasma of NZBWF1 mice. Autoreactive antibodies in plasma at sacrifice were measured by ELISA. Data are group mean ± SEM (n = 7–8/gp) and were analyzed by One-Way ANOVA on Ranks with Dunn’s method. Asterisk indicates statistically significant difference in antibody concentration between *c*SiO_2_ treatment and vehicle control (*p* < 0.05). *c*SiO_2_ dose significantly correlated (*p* < 0.05) with plasma anti-dsDNA Ab’s (Spearman rank-order correlation coefficient = 0.62) and anti-nuclear Ab’s (Spearman rank-order correlation coefficient = 0.58).

Marked elevations of total Ig anti-dsDNA antibodies (Fig 11A) and anti-nuclear antibodies (Fig 11B) were detected in plasma of *c*SiO_2_-dosed NZBWF1 mice compared to vehicle-treated mice. No changes in plasma anti-dsDNA antibodies were detected in C57Bl/6 mice exposed to *c*SiO_2_ or vehicle (S1 Fig). *c*SiO_2_ dose-dependently induced elevation of the proinflammatory cytokines MCP-1, TNF-α, and IL-6 (Fig 11D–11F) in NZBWF1 mice whereas effects on plasma cytokines IFN-γ, IL-10 or IL-12p70 were undetectable.

## Discussion

Human epidemiological findings support the contention that airway exposure to *c*SiO_2_ is etiologically linked to development of autoimmunity [4–6,39–43]. However, such studies shed little light on the mechanisms that underlie this putative association. Towards that end, we investigated the effects of short-term repeated *c*SiO_2_ exposure on autoimmune disease development in the NZBWF1 female mouse, a widely used experimental animal model for SLE. Several novel observations regarding this lupus-prone mouse strain were made 12 wk after the final *c*SiO_2_ exposure. First, *c*SiO_2_ triggered the onset of and exacerbated glomerulonephritis. Second, concurrent with kidney effects, *c*SiO_2_ induced marked lymphocyte infiltration in the lungs as well as dramatic increases in total immunoglobulins (IgG, IgA and IgM), and proinflammatory cytokine concentrations. Third, a striking observation in the lungs of NZBWF1 mice was the identification of extensive ELT that likely contributes to early triggering of SLE after *c*SiO_2_ exposure. Finally, in plasma, we found that *c*SiO_2_ exposure elicited marked elevations in autoreactive antibodies and proinflammatory cytokine concentrations. Collectively, these findings suggest intensive *c*SiO_2_ exposure via the airways over a discrete period evoked long-lasting injurious inflammatory and autoimmune responses that were evident at the site of exposure (lung), systemically, and at a distal tissue site (kidney).

Elevated plasma titers of autoreactive antibodies are an immunological hallmark for human SLE. Systemic autoantibodies form immune complexes with circulating self-antigens (e.g. dsDNA and nucleosome fractions). When deposited in the kidney [29,44–46], these immune complexes promote proinflammatory cytokine and chemokine production that mediate infiltration by mononuclear cells and consequent tissue injury [47]. Congruent with these mechanisms, heightened severity of glomerulonephritis was observed here in *c*SiO_2_-treated NZBWF1 mice concurrently with early onset of proteinuria. These findings are consistent with previous observations in *c*SiO_2_-exposed NZM2410 mice [29].

Several epidemiological studies have identified an association between occupational crystalline silica exposure and development of end-stage renal disease [39,48,49], particularly glomerulonephritis [50,51]. In a previous study by our laboratory, NZBWF1 mice fed diet identical to that employed here (safflower oil-based AIN-93G) spontaneously developed proteinuria at 30 wk of age [34]. Herein, NZBWF1 dosed with 1.0 mg *c*SiO_2_ exhibited proteinuria beginning at 22 wk of age. Thus, intranasal exposure to *c*SiO_2_ likely decreased the time to onset of lupus nephritis by 8 wk. Interestingly, kidney lesions were also increased in *c*SiO_2_-treated C57Bl/6 mice, albeit to a more modest extent. The observation of renal pathology consistent with lupus nephritis in C57Bl/6 mice supports the notion of an association between *c*SiO_2_ exposure and kidney disease. Consistent with these findings, autoimmune-like responses in kidneys including IgG deposition have been previously described after intratracheal administration of fibrogenic particles in C57Bl/6 mice [52,53]. While overt proteinuria (≥ 300 mg/dl) did not develop over the 12 wk duration of our study, it might be speculated that given sufficient time, short-term repeated airway exposure to *c*SiO_2_ might induce autoimmune nephritis in C57Bl/6 and other mice without evident genetic predisposition to SLE.

Although a prior study of *c*SiO_2_-induced pulmonary inflammation in NZM2410 autoimmune mice qualitatively described inflammatory cell infiltration in the lung [29], this report is the first to semi-quantitatively grade this response. A critical finding in this study was that NZBWF1 mice exposed to *c*SiO_2_ exhibited extensive lymphoplasmacytic infiltration in peribronchial and perivasculature regions of the lungs. Similar but more modest effects were observed in C57Bl/6 mice. This observation is consistent with the findings of Singh et al. [54] who suggested that the magnitude of perivasculature and peribronchial inflammation induced by pulmonary toxicant exposure is dependent on genetic background.

A critical question relates to the relevance of the doses employed in this study to actual human exposure. Using the NIOSH limit for respirable *c*SiO_2_ established at 0.05mg/m^3^/d and the assumption that human ventilation rate is 6.0L/min, we calculate that humans could be exposed to 1433mg silica in 40 years of work (8h/d for 5 d/wk) with respirable *c*SiO_2_. Based on the assumption that mice ventilation rate is 0.03L/min, we estimate that an equivalent lifetime exposure to crystalline silica would be 8.28 mg in mouse. Therefore, the two cumulative doses of *c*SiO_2_ used in this study, 1 and 4 mg, represent approximately one eighth and one half, respectively, of a human lifetime exposure to *c*SiO_2_ at the recommended NIOSH exposure limit.

We opted here to employ intranasal instillation of *c*SiO_2_ based on its successful use in previous studies with NZM2410 mice [29,30,32,35]. Other routes of *c*SiO_2_ administration in murine species have been described including inhalation, intratracheal and trans-oral instillation (oropharyngeal aspiration) [55–57]. Development of silicosis in four in-bred mouse strains, including lupus-prone MRL/MpJ mice, was assessed 16 wks after inhalation exposure to 70 mg m^-3^ *c*SiO_2_ for 5 h for 12 consecutive days [58]. MRL/MpJ mice developed extensive pulmonary lymphocytic infiltrates, similar to those observed herein with lupus-prone NZBWF1 mice, whereas Balb/c mice given the same exposure exhibited only mild pulmonary histopathological lesions.

Although inhalation exposure of rodent species is the gold standard for modeling human airway exposure to respirable toxicants such as crystalline silica, the need for specialized inhalation exposure chambers, rigorous technical considerations and high operational costs limit these experiments to only a few laboratory facilities. A comparative study of intratracheal, trans-oral, and intranasal instillation in Balb/c mice receiving a single 1.0 mg dose of *c*SiO_2_ revealed that each method was capable of eliciting significant pulmonary inflammation 4 h after exposure [59]. Intranasal instillation resulted in comparatively less inflammation relative to both trans-oral and intratracheal instillation. This could be attributed to trapping of *c*SiO_2_ particles in the nasal cavity, resulting in delayed passage to the lower airways [59]. However, this effect is not likely to be an issue in long-term studies of intranasal exposure to *c*SiO_2_, as mice treated in this manner eventually develop pulmonary fibrosis, a hallmark of *c*SiO_2_ exposure [35,60,61]. These latter studies support the contention that the high-dose, short-term repeated intranasal exposure employed here is a suitable model for investigations of environmentally triggered onset of autoimmunity in lupus-prone mice. Nevertheless, in the future it will be of interest to compare the effects of other routes of *c*SiO_2_ exposure, especially inhalation, on development of autoimmunity in NZBWF1 mice.

An unexpected observation in this study was extensive ectopic lymphoid neogenesis in the lung after *c*SiO_2_ exposure in NZBWF1 mice. ELT morphologically and functionally resemble secondary lymphoid tissues (e.g. lymph nodes) by supporting maturation and differentiation of B cells into plasma cells that produce class-switched antibodies [62]. ELT have been previously identified in target tissues of several autoimmune diseases, including the kidneys of aged NZWBF1 mice [63], synovial joints in rheumatoid arthritis [64,65], and salivary glands in Sjögren’s syndrome [66–68], where they contribute to local manifestations of autoimmunity. Although events that drive formation of ELT remain poorly defined, neogenesis of ectopic lymphoid structures is indicative of tissue-specific chronic inflammation [69] and importantly, may influence systemic autoimmunity [70]. Taken together, *c*SiO_2_-triggered formation of pulmonary ELT likely contributed to the exacerbated systemic autoimmunity observed in NZBWF1 mice following *c*SiO_2_ exposure.

Elevated BALF concentrations of TNF-α, MCP-1, and IL-6 observed here in *c*SiO_2_-exposed NZBWF1 mice are key observations because these cytokines play critical roles in both initiating innate and amplifying adaptive immune responses. The sustained elevation of these cytokines in BALF up to 12 wk after *c*SiO_2_ exposure suggests that the initial innate immune response was insufficient to contain the inflammation, supporting a previously described model for continuous alveolar macrophage apoptosis after *c*SiO_2_ exposure with ensuing self-antigen presentation [12]. Robust elevation of TNF-α in BALF has been similarly reported in *c*SiO_2_-exposed NZM2410 mice [30]. TNF-α induces apoptosis in alveolar macrophages and therefore could contribute to increased apoptotic debris containing autoantigens [12,31]. This cytokine can also stimulate production of additional proinflammatory cytokines, which could further amplify the immune response to *c*SiO_2_ [71–74]. Released predominately by monocytes/macrophages, MCP-1 recruits monocytes from systemic circulation to local sites of inflammation after tissue injury in attempt to contain or resolve further inflammation. The heightened elevation of this chemokine in BALF 12 wks following *c*SiO_2_ exposure further supports the notion that *c*SiO_2_ induces a continuous cycle of alveolar macrophage apoptosis, as sustained recruitment of circulating monocytes to the lungs by MCP-1 is likely an innate attempt to repopulate lost alveolar macrophages and resolve inflammation. We observed robust increases in the percent of lymphocytes in BALF recovered from *c*SiO_2_-treated NZBWF1 mice. Previous studies in cSiO_2_ exposed NZM2410 mice have indicated that both B and T cells contribute to increased lymphocytes in BALF [30]. IL-6 has a well-established role in both CD4+ T cell survival and B-cell differentiation to Ig-secreting plasma cells [75]. Taken together, the increase of lymphocytes in the presence of IL-6 in BALF likely promotes antigen-specific effector responses in the lungs. Indeed, in the lung, *c*SiO_2_-induced elevation of IL-6 corresponded to significant increases of IgG in BALF of NZBWF1 mice.

Mechanistic studies have indicated that alveolar macrophages are key to not only *c*SiO_2_-induced pulmonary pathogenesis [12], but are also essential for development of IgG immune complex-mediated inflammation in the lungs [76,77]. Following exposure to chrysotile asbestos, *in vitro* production of superoxide anion by alveolar macrophages was enhanced by stimulation with IgG [78]. Proinflammatory cytokines in lungs were markedly reduced in C57Bl/6 mice deficient in FcγRIII, which mediates activation of mononuclear phagocytes by binding the Fc region of the IgG molecule [79]. The notion that IgG both initiates and enhances lung inflammatory responses through the alveolar macrophage is therefore of possible significance to *c*SiO_2_ triggering of SLE in autoimmune-prone mice. Extensive deposition/production of IgG in lungs of *c*SiO_2_-exposed NZBWF1 mice could act synergistically with defective clearance of apoptotic macrophages, further contributing to acceleration of autoimmunity.

Airway exposure to *c*SiO_2_ stimulates alveolar macrophages, epithelial cells, and fibroblasts that mediate recruitment of circulating monocytes, neutrophils, and lymphocytes by releasing an array of inflammatory mediators including cytokines [57,80–82]. The close proximity of these inflammatory cells to both the airways and vasculature in this study suggest that these cells are not only capable of mediating production of proinflammatory mediators that impact the lung, but that may also be secreted into systemic circulation, thereby exacerbating development of systemic autoimmunity. It is notable that *c*SiO_2_-induced plasma increases of TNF-α and IL-6 mirrored elevations of these cytokines in BALF. Importantly, systemic concentrations of TNF-α and IL-6 correlate with SLE disease activity in humans [83] and treatment with exogenous IL-6 exacerbates glomerulonephritis in NZBWF1 mice [84]. There was also a trend towards elevated MCP-1 in plasma of NZBWF1 mice exposed to *c*SiO_2_. Urinary MCP-1 concentration has been identified as a biomarker of disease activity in lupus nephritis [85,86], and one study indicated that renal expression of MCP-1 correlates with NF-ΚB activation in kidney [87]. Overall, these results suggest that elevated plasma proinflammatory cytokines induced after *c*SiO_2_ exposure might further contribute to production of plasma autoantibodies as well as exacerbated renal pathology. Interestingly, cytokine array analysis of *c*SiO_2_-exposed NZM2410 mice failed to reveal any significant difference in plasma cytokines IL-4, IFN-γ, IL-10, IL-12, and TNF-α [30] suggesting some inherent differences in the response to intranasal *c*SiO_2_ might exist between that strain and the NZBWF1 employed here.

To summarize, the results presented here suggest that following airway exposure to *c*SiO_2_, the lung serves as a platform for the early triggering and exacerbation of systemic autoimmunity and glomerulonephritis in the NZBWF1 mouse. This model can serve as a starting point for further studies to gain insight into toxicant-triggered autoimmunity. First, it will be essential to characterize antigen-presenting cell and lymphocyte subpopulations recruited to and migrating out of the lung after *c*SiO_2_ exposure. These cells have the potential to drive subsequent tissue-specific homing of effector cell populations that mediate pathological outcomes in the lung and kidney. Second, while it is apparent that *c*SiO_2_ induces plasma elevation of proinflammatory cytokines, further studies are warranted to ascertain if these originate from lung ELT, inflamed kidneys, and/or immune tissues such as spleen. Third, this model of *c*SiO_2_-accelerated lupus can be used to study potential approaches for prevention and intervention in occupationally exposed human populations. A particularly attractive approach is the consumption of n-3 polyunsaturated fatty acids in fish oil which have been shown to delay onset and severity of autoimmune nephritis in NZBWF1 and other models [34,88]. Finally, the strategy described herein could be used to investigate whether other airborne toxicants that cause inflammatory responses in the lung might similarly exacerbate autoimmunity.

## Supporting Information

S1 Fig cSiO_2_ did not significantly alter anti-dsDNA Ab’s in plasma of C57Bl/6 mice. Antibodies in plasma at sacrifice were measured by ELISA. Data are mean ± SEM (n = 7–8/gp) and were analyzed by Mann-Whitney Rank Sum Test.
(TIFF)

## References

[1] National Institute of Allergy and Infectious Diseases (NIAID). Autoimmune Diseases Coordinating Committee. Progress in autoimmune diseases research. NIAID Report to Congress. 2005. Available: https://www.niaid.nih.gov/topics/autoimmune/Documents/adccfinal.pdf.

[2] Sacks JJ, Luo Y-H, Helmick CG. Prevalence of specific types of arthritis and other rheumatic conditions in the ambulatory health care system in the United States, 2001–2005. Arthritis Care Res. 2005;62: 460–464. 10.1002/acr.20041 20391499

[3] National Institude for Occupational Safety and Health. Health effects of occupational exposure to respirable crystalline silica. 2002. Available: http://www.cdc.gov/niosh/docs/2002-129/pdfs/2002-129.pdf

[4] Cooper GS, Parks CG. Occupational and environmental exposures as risk factors for systemic lupus erythematosus. Curr Rheumatol Rep. 2004;6: 367–374. 15355749 10.1007/s11926-004-0011-6

[5] Parks CG, Cooper GS, Nylander-French LA, Sanderson WT, Dement JM, Cohen PL, et al Occupational exposure to crystalline silica and risk of systemic lupus erythematosus—A population-based, case-control study in the southeastern United States. Arthritis Rheum. 2002;46: 1840–1850. 12124868 10.1002/art.10368

[6] Finckh A, Cooper GS, Chibnik LB, Costenbader KH, Watts J, Pankey H, et al Occupational silica and solvent exposures and risk of systemic lupus erythematosus in urban women. Arthritis Rheum. 2006;54: 3648–3654. 17075811 10.1002/art.22210

[7] Conrad K, Mehlhorn J, Luthke K, Dorner T, Frank K-H. Systemic lupus erythematosus after heavy exposure to quartz dust in uranium mines: clinical and serological characteristics. Lupus. 1996;5: 62–69. 8646229 10.1177/096120339600500112

[8] Rocha-Parise M, Santos LM, Damoiseaux JG, Bagatin E, Lido AV, Torello CO, et al Lymphocyte activation in silica-exposed workers. Int J Hyg Environ Health. 2014;217: 586–591. 10.1016/j.ijheh.2013.11.002 24332681

[9] Zaghi G, Koga F, Nisihara RM, Skare TL, Handar A, Rosa Utiyama SR, et al Autoantibodies in silicosis patients and in silica-exposed individuals. Rheumatol Int. 2010;30: 1071–1075. 10.1007/s00296-009-1116-z 19705119

[10] Albuquerque De Castro H, Gimenes Da Silva C, Lemle A. Immunoglobulins, complements and autoantibodies in 58 workers exposed to silica. Jornal Brasileiro de Pneumologia. 2003;30: 201–206.

[11] Doll NJ, Stankus RP, Hughes J, Weill H, Gupta RC, Rodriguez M et al Immune complexes and autoantibodies in silicosis. J Allergy Clin Immunol. 1981;68: 281–285. 6974746 10.1016/0091-6749(81)90152-4

[12] Hamilton RF Jr., Thakur SA, Holian A. Silica binding and toxicity in alveolar macrophages. Free Radic Biol Med. 2008;44: 1246–1258. 10.1016/j.freeradbiomed.2007.12.027 18226603 PMC2680955

[13] Hamilton RF Jr., Thakur SA, Mayfair JK, Holian A. MARCO mediates silica uptake and toxicity in alveolar macrophages from C57BL/6 mice. J Biol Chem. 2006;281: 34218–34226. 16984918 10.1074/jbc.M605229200

[14] Holian A, Uthman MO, Goltsova T, Brown SD, Hamilton RF Jr. Asbestos and silica-induced changes in human alveolar macrophage phenotype. Environ Health Perspect. 1997;105 Suppl 5: 1139–1142. 9400713 10.1289/ehp.97105s51139 PMC1470149

[15] Iyer R, Hamilton RF, Li L, Holian A. Silica-induced apoptosis mediated via scavenger receptor in human alveolar macrophages. Toxicol Appl Pharmacol. 1996;141: 84–92. 8917679 10.1006/taap.1996.0263

[16] Migliaccio CT, Hamilton RF Jr., Holian A. Increase in a distinct pulmonary macrophage subset possessing an antigen-presenting cell phenotype and in vitro APC activity following silica exposure. Toxicol Appl Pharmacol. 2005;205: 168–176. 15893544 10.1016/j.taap.2004.11.005

[17] Thakur SA, Beamer CA, Migliaccio CT, Holian A. Critical role of MARCO in crystalline silica-induced pulmonary inflammation. Toxicol Sci. 2009;108: 462–471. 10.1093/toxsci/kfp011 19151164 PMC2664690

[18] Chen F, Sun SC, Kuh DC, Gaydos LJ, Demers LM. Essential role of NF-kappa B activation in silica-induced inflammatory mediator production in macrophages. Biochem Biophys Res Commun. 1995;214: 985–992. 7575573 10.1006/bbrc.1995.2383

[19] Di Giuseppe M, Gambelli F, Hoyle GW, Lungarella G, Studer SM, Richards T, et al Systemic inhibition of NF-KB activation protects from silicosis. PLoS ONE. 2009;4: e5689 10.1371/journal.pone.0005689 19479048 PMC2682759

[20] Piguet PF, Collart MA, Grau GE, Sappino AP, Vassalli P. Requirement of tumour necrosis factor for development of silica-induced pulmonary fibrosis. Nature. 1990;344: 245–247. 2156165 10.1038/344245a0

[21] Gabay C, Cakir N, Moral F, Roux-Lombard P, Meyer O, Dayer JM, et al Circulating levels of tumor necrosis factor soluble receptors in systemic lupus erythematosus are significantly higher than in other rheumatic diseases and correlate with disease activity. J Rheumatol. 1997;24: 303–308. 9034987

[22] Davas EM, Tsirogianni A, Kappou I, Karamitsos D, Economidou I, Dantis PC, et al Serum IL-6, TNFalpha, p55 srTNFalpha, p75srTNFalpha, srIL-2alpha levels and disease activity in systemic lupus erythematosus. Clin Rheumatol. 1999;18: 17–22. 10088943 10.1007/s100670050045

[23] Brown JM, Pfau JC, Pershouse MA, Holian A. Silica, apoptosis, and autoimmunity. J Immunotoxicol. 2005;1: 177–187. 10.1080/15476910490911922 18958651

[24] Rudofsky UH, Lawrence DA. New Zealand mixed mice: a genetic systemic lupus erythematosus model for assessing environmental effects. Environ Health Perspect. 1999;107 Suppl 5: 713–721. 10502536 10.1289/ehp.99107s5713 PMC1566260

[25] Perry D, Sang A, Yin Y, Zheng YY, Morel L. Murine models of systemic lupus erythematosus. J Biomed Biotechnol. 2011;2011: 271694 10.1155/2011/271694 21403825 PMC3042628

[26] Sang A, Yin Y, Zheng YY, Morel L. Animal models of molecular pathology systemic lupus erythematosus. Prog Mol Biol Transl Sci. 2012;105: 321–370. 10.1016/B978-0-12-394596-9.00010-X 22137436

[27] Mohan C, Yu Y, Morel L, Yang P, Wakeland EK. Genetic dissection of Sle pathogenesis: Sle3 on murine chromosome 7 impacts T cell activation, differentiation, and cell death. J Immunol. 1999;162: 6492–6502. 10352264

[28] Rudofsky UH, Evans BD, Balaban SL, Mottironi VD, Gabrielsen AE. Differences in expression of lupus nephritis in New Zealand mixed H-2z homozygous inbred strains of mice derived from New Zealand black and New Zealand white mice. Lab Invest. 1993;68: 419–426. 8479150

[29] Brown JM, Archer AI, Pfau IC, Holian A. Silica accelerated systemic autoimmune disease in lupus-prone New Zealand mixed mice. Clin Exp Immunol. 2003;131: 415–421. 12605693 10.1046/j.1365-2249.2003.02094.x PMC1808650

[30] Brown JM, Pfau JC, Holian A. Immunoglobulin and lymphocyte responses following silica exposure in New Zealand mixed mice. Inhal Toxicol. 2004;16: 133–139. 15204774 10.1080/08958370490270936

[31] Pfau JC, Brown JM, Holian A. Silica-exposed mice generate autoantibodies to apoptotic cells. Toxicology. 2004;195: 167–176. 14751672 10.1016/j.tox.2003.09.011

[32] Brown JM, Schwanke CM, Pershouse MA, Pfau JC, Holian A. Effects of rottlerin on silica-exacerbated systemic autoimmune disease in New Zealand mixed mice. Am J Physiol Lung Cell Mol Physiol. 2005;289: L990–998. 16040631 10.1152/ajplung.00078.2005

[33] Berthier CC, Bethunaickan R, Gonzalez-Rivera T, Nair V, Ramanujam M, Zhang W, et al Cross-species transcriptional network analysis defines shared inflammatory responses in murine and human lupus nephritis. J Immunol. 2002;189: 988–1001. 10.4049/jimmunol.1103031 PMC3392438 22723521

[34] Pestka JJ, Vines LL, Bates MA, He K, Langohr I. Comparative effects of n-3, n-6 and n-9 unsaturated fatty acid-rich diet consumption on lupus nephritis, autoantibody production and CD4+ T cell-related gene responses in the autoimmune NZBWF1 Mouse. PLoS ONE. 2014;9: e100255 10.1371/journal.pone.0100255 24945254 PMC4063768

[35] Beamer CA, Migliaccio CT, Jessop F, Trapkus M, Yuan D, Holian A. Innate immune processes are sufficient for driving silicosis in mice. J Leukoc Biol. 2010;88: 547–557. 10.1189/jlb.0210108 20576854 PMC2924603

[36] Brandenberger C, Rowley NL, Jackson-Humbles DN, Zhang Q, Bramble LA, Lewandowski RP, et al Engineered silica nanoparticles act as adjuvants to enhance allergic airway disease in mice. Part Fibre Toxicol. 2013;10: 26 10.1186/1743-8977-10-26 23815813 PMC3729411

[37] Weening JJ, D'Agati VD, Schwartz MM, Seshan SV, Alpers CE, Appel GB, et al The classification of glomerulonephritis in systemic lupus erythematosus revisited. Kidney Int. 2004;65: 521–530. 14717922 10.1111/j.1523-1755.2004.00443.x

[38] Yan D, Zhou HR, Brooks KH, Pestka JJ. Role of macrophages in elevated IgA and IL-6 production by Peyer's patch cultures following acute oral vomitoxin exposure. Toxicol Appl Pharmacol. 1998;148: 261–273. 9473534 10.1006/taap.1997.8326

[39] Steenland K, Sanderson W, Calvert GM. Kidney disease and arthritis in a cohort study of workers exposed to silica. Epidemiology. 2001;12: 405–412. 11416778 10.1097/00001648-200107000-00010

[40] Makol A, Reilly MJ, Rosenman KD. Prevalence of connective tissue disease in silicosis (1985–2006)—a report from the state of michigan surveillance system for silicosis. Am J Ind Med. 2011;54: 255–262. 10.1002/ajim.20917 20957678

[41] Speck-Hernandez CA, Montoya-Ortiz G. Silicon, a possible link between environmental exposure and autoimmune diseases: the case of rheumatoid arthritis. Arthritis. 2012;2012: 1–11. 23119159 10.1155/2012/604187 PMC3483651

[42] Steenland NK, Thun MJ, Ferguson CW, Port FK. Occupational and other exposures associated with male end-stage renal disease: a case/control study. Am J Public Health. 1990;80: 153–157. 2153349 10.2105/ajph.80.2.153 PMC1404604

[43] Rosenman KD, Moore-Fuller M, Reilly MJ. Connective tissue disease and silicosis. Am J Ind Med. 1999;35: 375–381. 10086214 10.1002/(sici)1097-0274(199904)35:4<375::aid-ajim8>3.0.co;2-i

[44] Clynes R, Dumitru C, Ravetch JV. Uncoupling of immune complex formation and kidney damage in autoimmune glomerulonephritis. Science. 1998;279: 1052–1054. 9461440 10.1126/science.279.5353.1052

[45] Gonzalez ML, Waxman FJ. Glomerular deposition of immune complexes made with IgG2a monoclonal antibodies. J Immunol. 2000;164: 1071–1077. 10623858 10.4049/jimmunol.164.2.1071

[46] Su W, Madaio MP. Recent advances in the pathogenesis of lupus nephritis: autoantibodies and B Cells. Semin Nephrol. 2003;23: 564–568. 14631564 10.1053/s0270-9295(03)00135-9

[47] Nowling TK, Gilkeson GS. Mechanisms of tissue injury in lupus nephritis. Arthritis Res Ther. 2001;13: 250–258. 10.1186/ar3528 PMC3334648 22192660

[48] Vupputuri S, Parks CG, Nylander-French LA, Owen-Smith A, Hogan SL, Sandler DP et al Occupational silica exposure and chronic kidney disease. Ren Fail. 2012;34: 40–46. 10.3109/0886022X.2011.623496 22032652 PMC3266824

[49] Steenland K. One agent, many diseases: exposure-response data and comparative risks of different outcomes following silica exposure. Am J Ind Med. 2005;48: 16–23. 15940719 10.1002/ajim.20181

[50] Calvert GM, Steenland K, Palu S. End-stage renal disease among silica-exposed gold miners. A new method for assessing incidence among epidemiologic cohorts. J Am Med Assoc. 1997;277: 1219–1223. 9103346

[51] Steenland K, Rosenman K, Socie E, Valiante D. Silicosis and end-stage renal disease. Scand J Work Environ Health. 2002;28: 439–442. 12539804 10.5271/sjweh.696

[52] Pfau JC, Sentissi JJ, Li S, Calderon-Garciduenas L, Brown JM, Blake DJ, et al Asbestos-induced autoimmunity in C57BL/6 mice. J Immunotoxicol. 2008;5: 129–137. 10.1080/15476910802085756 18569382

[53] Zebedeo CN, Davis C, Pena C, Ng KW, Pfau JC. Erionite induces production of autoantibodies and IL-17 in C57BL/6 mice. Toxicol Appl Pharmacol. 2014;275: 257–264. 10.1016/j.taap.2014.01.018 24518925 PMC4010586

[54] Singh B, Shinagawa K, Taube C, Gelfand EW, Pabst R. Strain-specific differences in perivascular inflammation in lungs in two murine models of allergic airway inflammation. Clin Exp Immunol. 2005;141: 223–229. 15996186 10.1111/j.1365-2249.2005.02841.x PMC1809429

[55] Gossart S, Cambon C, Orfila C, Seguelas MH, Lepert JC, Rami J, et al Reactive oxygen intermediates as regulators of TNF-alpha production in rat lung inflammation induced by silica. J Immunol. 1996;156: 1540–1548. 8568258

[56] Lakatos HF, Burgess HA, Thatcher TH, Redonnet MR, Hernady E, Williams JP, et al Oropharyngeal aspiration of a silica suspension produces a superior model of silicosis in the mouse when compared to intratracheal instillation. Exp Lung Res. 2006;32: 181–199. 16908446 10.1080/01902140600817465 PMC10208218

[57] Liu F, Liu J, Weng D, Chen Y, Song L, He Q, et al CD4+CD25+Foxp3+ regulatory T cells depletion may attenuate the development of silica-induced lung fibrosis in mice. PLoS One. 2010;5: e15404 10.1371/journal.pone.0015404 21072213 PMC2972313

[58] Davis GS, Leslie KO, Hemenway DR. Silicosis in mice: effects of dose, time, and genetic strain. J Environ Pathol Toxicol Oncol. 1998;17: 81–97. 9546745

[59] Lacher SE, Johnson C, Jessop F, Holian A, Migliaccio CT. Murine pulmonary inflammation model: a comparative study of anesthesia and instillation methods. Inhal Toxicol. 2010;22: 77–83. 10.3109/08958370902929969 20017595 PMC4068398

[60] Beamer CA, Holian A. Scavenger receptor class A type I/II (CD204) null mice fail to develop fibrosis following silica exposure. Am J Physiol Lung Cell Mol Physiol. 2005;289: L186–195. 15849212 10.1152/ajplung.00474.2004

[61] Ferreira TP, de Arantes AC, do Nascimento CV, Olsen PC, Trentin PG, Rocco PR, et al IL-13 immunotoxin accelerates resolution of lung pathological changes triggered by silica particles in mice. J Immunol. 2013;191: 5220–5229. 10.4049/jimmunol.1203551 24133168

[62] Dieu-Nosjean MC, Goc J, Giraldo NA, Sautes-Fridman C, Fridman WH. Tertiary lymphoid structures in cancer and beyond. Trends Immunol. 2014;35: 571–580. 10.1016/j.it.2014.09.006 25443495

[63] Schiffer L, Kumpers P, Davalos-Misslitz AM, Haubitz M, Haller H, Anders HJ, et al B-cell-attracting chemokine CXCL13 as a marker of disease activity and renal involvement in systemic lupus erythematosus (SLE). Nephrol Dial Transplant. 2009;24: 3708–3712. 10.1093/ndt/gfp343 19602475

[64] Takemura S, Klimiuk PA, Braun A, Goronzy JJ, Weyand CM. T cell activation in rheumatoid synovium is B cell dependent. J Immunol. 2001;167: 4710–4718. 11591802 10.4049/jimmunol.167.8.4710

[65] Wengner AM, Hopken UE, Petrow PK, Hartmann S, Schurigt U, Brauer R et al CXCR5- and CCR7-dependent lymphoid neogenesis in a murine model of chronic antigen-induced arthritis. Arthritis Rheum. 2007;56: 3271–3283. 17907173 10.1002/art.22939

[66] Hansen A, Lipsky PE, Dorner T. B cells in Sjogren's syndrome: indications for disturbed selection and differentiation in ectopic lymphoid tissue. Arthritis Res Ther. 2007;9: 218 17697366 10.1186/ar2210 PMC2206371

[67] Bombardieri M, Barone F, Lucchesi D, Nayar S, van den Berg WB, Proctor G, et al Inducible tertiary lymphoid structures, autoimmunity, and exocrine dysfunction in a novel model of salivary gland inflammation in C57BL/6 mice. J Immunol. 2012;189: 3767–3776. 22942425 10.4049/jimmunol.1201216 PMC3448973

[68] Stott DI, Hiepe F, Hummel M, Steinhauser G, Berek C. Antigen-driven clonal proliferation of B cells within the target tissue of an autoimmune disease. The salivary glands of patients with Sjogren's syndrome. J Clin Invest. 1998;102: 938–946. 9727062 10.1172/JCI3234 PMC508959

[69] Grogan JL, Ouyang W. A role for Th17 cells in the regulation of tertiary lymphoid follicles. Eur J Immunol. 2012;42: 2255–2262. 10.1002/eji.201242656 22949324

[70] Humby F, Bombardieri M, Manzo A, Kelly S, Blades MC, Kirkham B, et al Ectopic Lymphoid Structures Support Ongoing Production of Class-Switched Autoantibodies in Rheumatoid Synovium. Plos Medicine. 2009;6: 59–75. 10.1371/journal.pmed.0060001 PMC2621263 19143467

[71] Satriano J, Schlondorff D. Activation and attenuation of transcription factor NF-kB in mouse glomerular mesangial cells in response to Tumor Necrosis Factor-alpha, Immunoglobulin G, and adenosine 3':5'-cyclic monophosphate. J Clin Invest. 1994;94: 1629–1636. 7929839 10.1172/JCI117505 PMC295323

[72] Sanz AB, Sanchez-Nino MD, Ramos AM, Moreno JA, Santamaria B, Ruiz-Ortega M, et al NF-kappaB in renal inflammation. J Am Soc Nephrol. 2010;21: 1254–1262. 10.1681/ASN.2010020218 20651166

[73] Zheng L, Sinniah R, Hsu SI. Pathogenic role of NF-kappaB activation in tubulointerstitial inflammatory lesions in human lupus nephritis. J Histochem Cytochem. 2008;56: 517–529. 10.1369/jhc.7A7368.2008 18285351 PMC2324188

[74] Dostert C, Petrilli V, Van Bruggen R, Steele C, Mossman BT, Tschopp J, et al Innate immune activation through Nalp3 inflammasome sensing of asbestos and silica. Science. 2008;320: 674–677. 10.1126/science.1156995 18403674 PMC2396588

[75] Maeda K, Mehta H, Drevets DA, Coggeshall KM. IL-6 increases B-cell IgG production in a feed-forward proinflammatory mechanism to skew hematopoiesis and elevate myeloid production. Blood. 2010;115: 4699–4706. 10.1182/blood-2009-07-230631 20351305 PMC3790945

[76] Lentsch AB, Czermak BJ, Bless NM, Ward PA. NF-kappaB activation during IgG immune complex-induced lung injury: requirements for TNF-alpha and IL-1beta but not complement. Am J Pathol. 1998;152: 1327–1336. 9588901 PMC1858598

[77] Johnson KJ, Ward PA. Acute immunologic pulmonary alveolitis. J Clin Invest. 1974;54: 349–357. 4277008 10.1172/JCI107770 PMC301562

[78] Scheule RK, Holian A. IgG specifically enhances chrysotile asbestos-stimulated superoxide anion production by the alveolar macrophage. Am J Respir Cell Mol Biol. 1989;1: 313–318. 2560395 10.1165/ajrcmb/1.4.313

[79] Chouchakova N, Skokowa J, Baumann U, Tschernig T, Philippens KM, Nieswandt B, et al Fc gamma RIII-mediated production of TNF-alpha induces immune complex alveolitis independently of CXC chemokine generation. J Immunol. 2001;166: 5193–5200. 11290803 10.4049/jimmunol.166.8.5193

[80] Davis GS, Leslie KO, Schwarz JE, Pfeiffer LM, Hill-Eubanks L, Hemenway DR, et al Altered patterns of lung lymphocyte accumulation in silicosis in cytokine-sufficient (C3H/HeN) and cytokine-deficient (C3H/HeJ-LPSd) mice. Chest. 1993;103: 120s–121s. 8428530

[81] Davis GS, Holmes CE, Pfeiffer LM, Hemenway DR. Lymphocytes, lymphokines, and silicosis. J Environ Pathol Toxicol Oncol. 2001;20 Suppl 1: 53–65. 11570674

[82] Mossman BT, Churg A. Mechanisms in the pathogenesis of asbestosis and silicosis. Am J Respir Crit Care Med. 1998;157: 1666–1680. 9603153 10.1164/ajrccm.157.5.9707141

[83] Sabry A, Sheashaa H, El-Husseini A, Mahmoud K, Eldahshan KF, George SK, et al Proinflammatory cytokines (TNF-alpha and IL-6) in Egyptian patients with SLE: its correlation with disease activity. Cytokine. 2006;35: 148–153. 17027280 10.1016/j.cyto.2006.07.023

[84] Ryffel B, Car BD, Gunn H, Roman D, Hiestand P, Mihatsch MJ, et al Interleukin 6 exacerbates glomerulonephritis in (NZBXNZW)F-1 mice. Am J Pathol. 1994;144: 927–937. 8178944 PMC1887352

[85] Abujam B, Cheekatla S, Aggarwal A. Urinary CXCL-10/IP-10 and MCP-1 as markers to assess activity of lupus nephritis. Lupus. 2013;22: 614–623. 10.1177/0961203313484977 23629827

[86] Barbado J, Martin D, Vega L, Almansa R, Goncalves L, Nocito M, et al MCP-1 in urine as biomarker of disease activity in Systemic Lupus Erythematosus. Cytokine. 2012;60: 583–586. 10.1016/j.cyto.2012.07.009 22857869

[87] Rovin BH, Dickerson JA, Tan LC, Hebert CA. Activation of nuclear factor-KB correlates with MCP-1 expression by human mesangial cells. Kidney Int. 1995;48: 1263–1271. 8569088 10.1038/ki.1995.410

[88] Pestka JJ. n-3 Polyunsaturated fatty acids and autoimmune-mediated glomerulonephritis. Prost Leukotriene Essent Fatty Acids. 2010;82: 251–258. 10.1016/j.plefa.2010.02.013 20189790 PMC2885141

