# Supplementary figures and images for: Silica Triggers Inflammation and Ectopic Lymphoid Neogenesis in the Lungs in Parallel with Accelerated Onset of Systemic Autoimmunity and Glomerulonephritis in the Lupus-Prone NZBWF1 Mouse
Source: PLoS One. 2015 May 15;10(5):e0125481. doi: 10.1371/journal.pone.0125481 (PMC4433215; doi:10.1371/journal.pone.0125481)

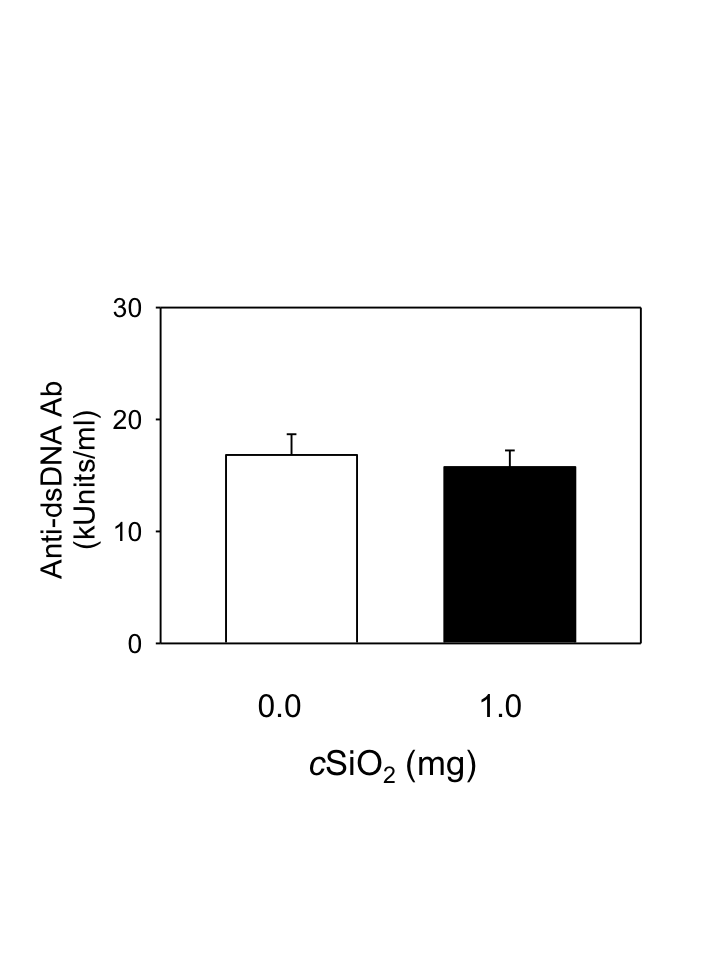

Supplement: S1 Fig — Antibodies in plasma at sacrifice were measured by ELISA. Data are mean ± SEM (n = 7–8/gp) and were analyzed by Mann-Whitney Rank Sum Test. (TIFF) [file pone.0125481.s001.tiff]
